# FIBP knockdown attenuates growth and enhances chemotherapy in colorectal cancer via regulating GSK3β-related pathways

**DOI:** 10.1038/s41389-018-0088-9

**Published:** 2018-10-02

**Authors:** Yan-Feng Huang, Wen-Bo Niu, Rong Hu, Ling-Jun Wang, Zeng-Yan Huang, Shi-Hao Ni, Ming-Qing Wang, Yi Yang, Yu-Sheng Huang, Wen-Jun Feng, Wei Xiao, Da-Jian Zhu, Shao-Xiang Xian, Lu Lu

**Affiliations:** 1 The First Affiliated Hospital, Guangzhou University of Chinese Medicine, 510407 Guangzhou, Guangdong China; 2 Shunde Hospital (The first People’s Hospital of Shunde Foshan), Southern Medical University, 528300 Foshan, China; 3 Cancer Research Institute, Southern Medical University, 510515 Guangzhou, China; 4 School of Traditional Chinese Medicine, Southern Medical University, 510515 Guangzhou, China; 5 Lingnan Medical Research Center, Guangzhou University of Chinese Medicine, 510407 Guangzhou, Guangdong China; 6 Department of Gastrointestinal Surgery, Guangdong Medical University Affiliated Women and Children Hospital, 528300 Foshan, China

## Abstract

Colorectal cancer stem cells (CSCs), characterized by self-renewal ability and high expression of proliferative genes, contribute to the chemoresistance of colorectal cancer (CRC). We aimed to identify the molecular mechanisms underlying CRC chemoresistance through comprehensive bioinformatics screenings and experimental confirmation of gene functions. We found that high expression of FGF1 intracellular binding protein (FIBP) was correlated with chemoresistance and poor prognosis in CRC patients. Therefore, the chemoresistant CRC cell line HCT116-CSC with high expression of the stem cell markers CD44 and CD133 was established for further phenotypic tests. FIBP knockdown inhibited proliferation, enhanced chemotherapy effects, and attenuated the stemness markers of CRC cells in vivo and in vitro. Through RNA-seq and gene set enrichment analysis, we identified cyclin D1 as a key downstream target in FIBP-regulated cell cycle progression and proliferation. Moreover, FIBP bound to GSK3β, inhibited its phosphorylation at Tyr216, and activated β-catenin/TCF/cyclin D1 signaling in HCT116-CSCs. Additional GSK3β knockdown reversed the FIBP silencing-induced inhibition of proliferation and decreased stemness marker expression in HCT116-CSCs. Furthermore, DNA methylation profiling suggested that FIBP regulated the stemness of CRC cells via methylation activity that was dependent on GSK3β but independent of β-catenin signaling. Our data illuminate the potential of FIBP as a novel therapeutic target for treating chemoresistant CRC through inhibition of GSK3β-related signaling.

## Introduction

Colorectal cancer (CRC) is the second leading cause of cancer-related death in the United States and the fifth in China^1,2^. Remarkably, the 5-year survival rate of patients with CRC metastasis is <10%^2^. Significant improvements in patient survival rates have been achieved in recent years, largely due to the contributions of antineoplastic chemotherapy. Although chemotherapy is effective for some metastatic CRC, these treatments often lead to dose-dependent enterotoxigenesis and acquired chemoresistance (CR)^3^. Currently, emerging evidence suggests the critical roles of colorectal cancer stem cells (CSCs) in conferring therapeutic resistance^4^. These stem-like cells are capable of self-renewal and are essential for initiating tumor formation and maintaining long-term tumor heterogeneity^5^. In the clinic, CRC patients with higher levels of stem cell-like makers associated with higher relapse and lower survival rates^6–8^. Therefore, novel therapeutic approaches targeted at overcoming drug resistance are urgently needed to improve CRC treatment.

Our previous studies showed that curcumin, a plant extract with antitumor activity, enhances the effects of irinotecan on CRC cell apoptosis through reactive oxygen species generation, activation of endoplasmic reticulum stress, and autophagy restoration^9,10^. Natural products tend to exert effects through multiple targets, and thus we explored potential targets of curcumin using a comparative proteomic analysis^11^. Fibroblast growth factor 1 (FGF1) intracellular binding protein (FIBP) was identified as a potential target molecule of curcumin treatment in irinotecan-induced apoptosis of CRC LOVO cells^11^. FIBP is an intracellular protein that binds selectively to acidic fibroblast growth factor (aFGF), which is mitogenic for a variety of cell types by stimulating mitogenesis or inducing morphological changes and differentiation. Moreover, FGFs and their chaperone molecules have been reported to participate in cancer development^12,13^.

FIBP increases tumorigenicity and is highly expressed in tumors, such as colon carcinoma^13,14^. The effects of altered FIBP expression on CRC phenotype are of great interest to us, especially with regard to the proliferation potential and stemness properties of CSCs because, currently, whether and how FIBP regulates CRC CR remains largely elusive. Thus we performed in vivo and in vitro experiments to examine cellular phenotypes and explored the underlying mechanisms of FIBP-regulated CRC cell proliferation, which provided useful insight into the targeting of FIBP for effective treatment of chemoresistant CRC.

## Results

### FIBP is a prognostic indicator of CRC progression and exhibited high expression in CRC tissues from chemoresistant patients

**Fig. 1:**
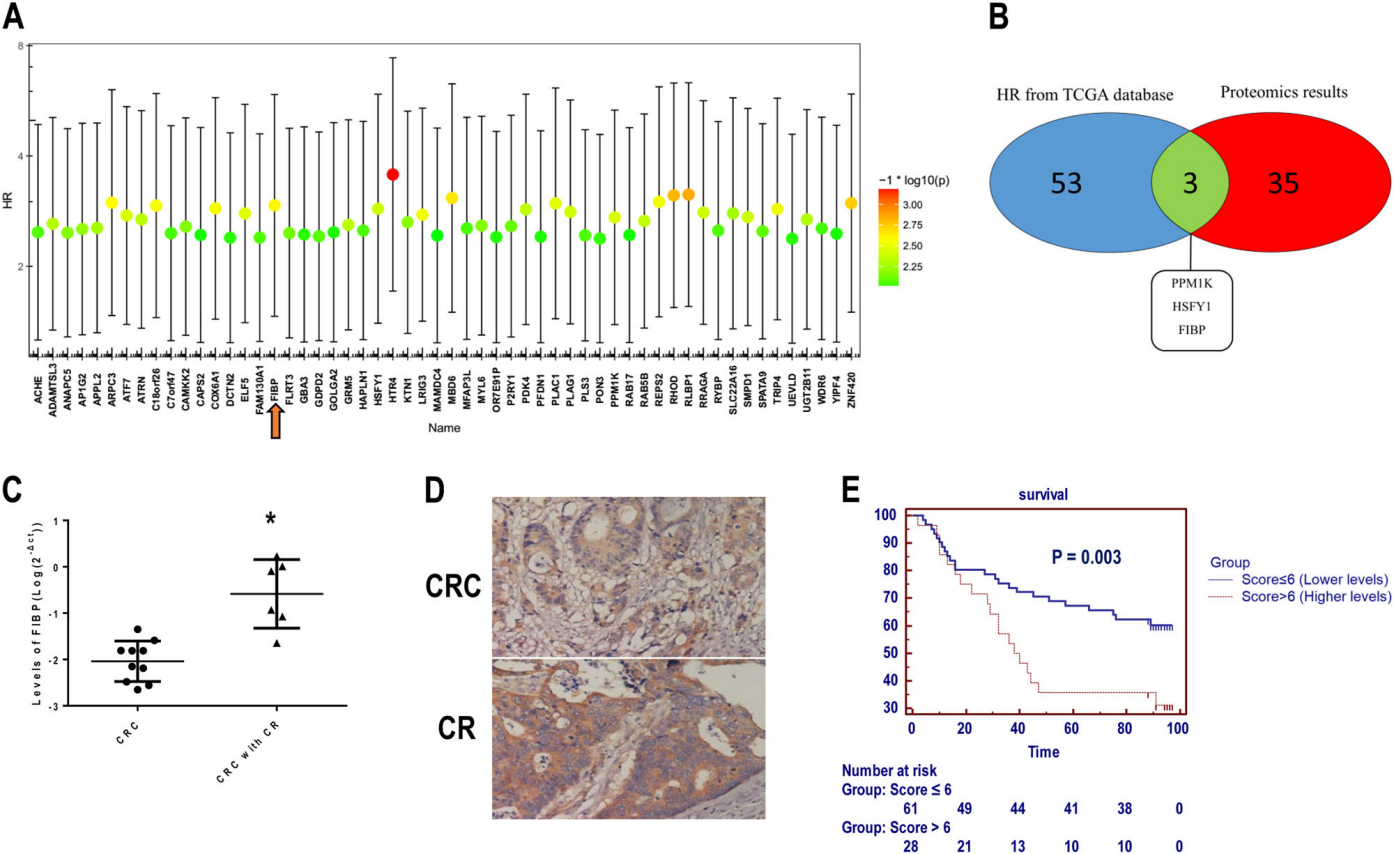
FIBP is a prognostic indicator of CRC progression and is expressed at higher levels in chemoresistant CRC tissues. **a** The hazard risk (HR) distribution of 56 genes with high expression and association with poor prognosis based on TCGA raw data from cBioPortal. The filtering criteria are *p* < 0.05 and HR > 0. **b** Venn diagram showing that FIBP, HSFY1, and PPM1K are simultaneously present in both the set of genes with a high HR and the set of genes downregulated by curcumin. **c** Expression of FIBP was higher in CRC specimens with chemoresistance (CR) than in chemosensitive tissue. *n* = 10 for the CRC group, and *n* = 6 for the CRC with CR group. **d** Representative immunochemistry staining results in tumors with/without chemoresistance (magnification, ×200). **e** Kaplan–Meier survival curves according to FIBP levels. *p < 0.05 CRC vs. CRC with CR

Selection of the most beneficial treatment regimens in CRC remains a challenge and is hindered by a lack of predictive and prognostic markers^15^. First, we performed a comprehensive bioinformatics evaluation of 17,814 genes to determine potential markers by calculating hazard risk (HR) based on the raw survival data and gene expression data from The Cancer Genome Atlas Colorectal Adenocarcinoma. The cutoff value was based on the median expression of individual genes. Overall, 56 genes passed the filtering criteria (*p* < 0.05 and HR > 0, Fig. 1a). The genes with higher expression were considered to be of prognostic value and associated with poor prognosis in CRC. In combination with our previous proteomic analysis results, a Venn diagram showed that three genes (FIBP, HSFY1, PPM1K) are simultaneously present in both the group of genes with a high HR and the group of genes downregulated by curcumin (Fig. 1b). Although FIBP shows a high HR for CRC progression (HR = 2.933, 95% confidence interval (CI) = 1.462–5.884, *p* = 0.002), little is currently known regarding the association between FIBP and CRC progression. Taking into account that curcumin was able to improve CR in our previous work^9–11^, we quantitated FIBP expression in CRC specimens with or without CR. Notably, the transcript levels of FIBP were significantly higher in CRC specimens with CR than in those without CR (*p* = 0.002, Fig. 1c), and this was validated by subsequent immunochemistry analysis (Fig. 1d). Furthermore, we analyzed FIBP expression using human tissue microarray slides derived from CRC patients. Our data showed that the relative risk of death associated with high FIBP expression (score >6) was 2.696 (95% CI = 1.407–5.166, *p* = 0.003; Fig. 1e). After adjustment for other clinical parameters, including age, gender, lymph node metastasis, tumor size, pathological grade, American Joint Committee on Cancer (AJCC) stage, and Tumor Node Metastasis (TNM) stage, the results still displayed statistical significance (HR = 2.988, 95% CI = 1.512–5.903, *p* = 0.002; Table S2).

### FIBP knockdown inhibited proliferation of CRC cells in vivo and in vitro

**Fig. 2:**
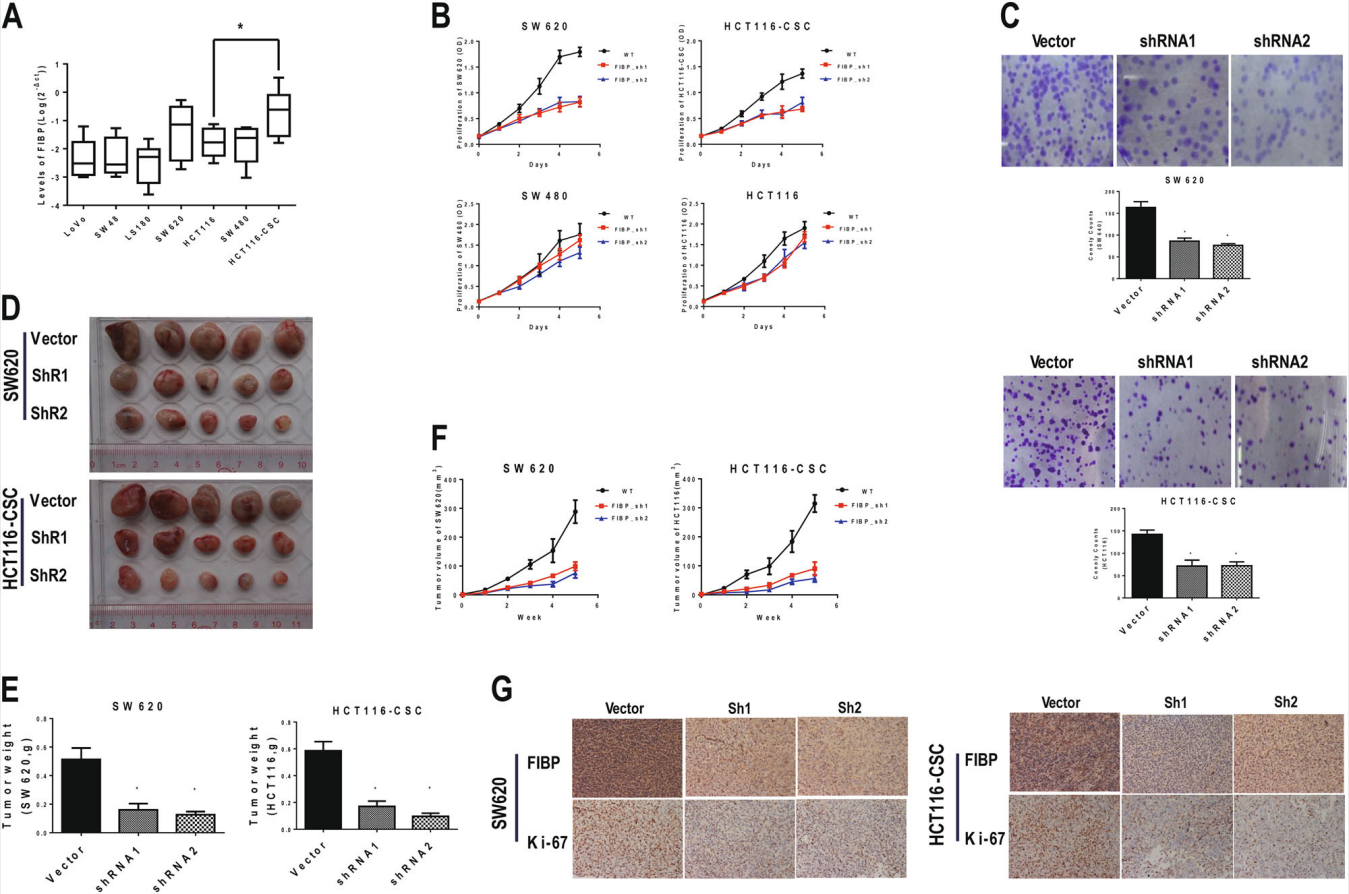
FIBP knockdown inhibited proliferation of CRC cells in vitro and in vivo. **a** The expression of FIBP in various CRC cell lines was quantitated by qPCR. HCT116-CSC is the CSC-enriched chemoresistant cell line derived from parental HCT116 cells. *n* = 8 for each tested cell line. **b** Proliferation curves of CRC SW620, SW480, and HCT116 and HCT116-CSC cell lines with or without FIBP knockdown. **c** Colony-formation assays of SW620 cells and HCT116-CSCs with control or FIBP-shRNA lentivirus infection. **d**–**g** Nude mice were inoculated with SW620 cells or HCT116-CSCs pre-infected with control or FIBP-shRNA lentivirus and were sacrificed 5 weeks later. Images (**d**) and weight (**e**) of CRC xenograft tumors are shown. **f** Growth of xenograft tumors derived from SW620 cells and HCT116-CSCs with or without FIBP knockdown. **g** Respective immunochemistry staining of FIBP and Ki67 expression in tumors with or without FIBP knockdown (magnification, ×200). *p < 0.05, compared with the Vector control group

Next, we examined FIBP expression in six common CRC cell lines. FIBP levels in SW620, SW480, and HCT116 cells were significantly higher than in the other three cell lines (LoVo, SW48, and LS180) and in two normal colonic epithelial cell lines (NCM460 and FHC). Notably, HCT116-CSC, the chemoresistant CSC-enriched HCT-116 cell line we established (Figure S1 and 2), displayed the highest FIBP expression (Fig. 2a and Figure S3). FIBP knockdown in all three of the cell lines with higher FIBP expression exhibited significant growth inhibition at day 5 compared with the control cells (Fig. 2b). Particularly, SW620 cells and HCT116-CSCs displayed more proliferation inhibition (*p* < 0.001) than SW480 (*p* = 0.004) and HCT116 (*p* = 0.008) cells at day 5, likely due to the lower FIBP expression levels in these two cell lines or lower viral transfection efficiency. Furthermore, colony-formation assays indicated that FIBP knockdown significantly decreased the number of SW620 (*p* < 0.01) and HCT116-CSC (*p* < 0.01) cell colonies (Fig. 2c). Moreover, our results suggested that FIBP controlled CRC cell growth in part by regulating cell apoptosis and migration (Figure S4). To investigate the effect of FIBP silencing on tumor growth in vivo, we established xenograft mouse models by injecting SW620 cells and HCT116-CSCs with/without FIBP knockdown into the left/right dorsal flank of mice, respectively. Consistently, FIBP knockdown significantly decreased xenograft tumor burden (Fig. 2d), as evidenced by reduced tumor weight (Fig. 2e) and tumor size during the observation time of 5 weeks (Fig. 2f) for both SW620 cells and HCT116-CSCs. Furthermore, IHC staining demonstrated significantly decreased Ki-67 levels in tumors with FIBP knockdown (Fig. 2g).

### FIBP fostered G1–S-phase transition and activated cell cycle signaling

**Fig. 3:**
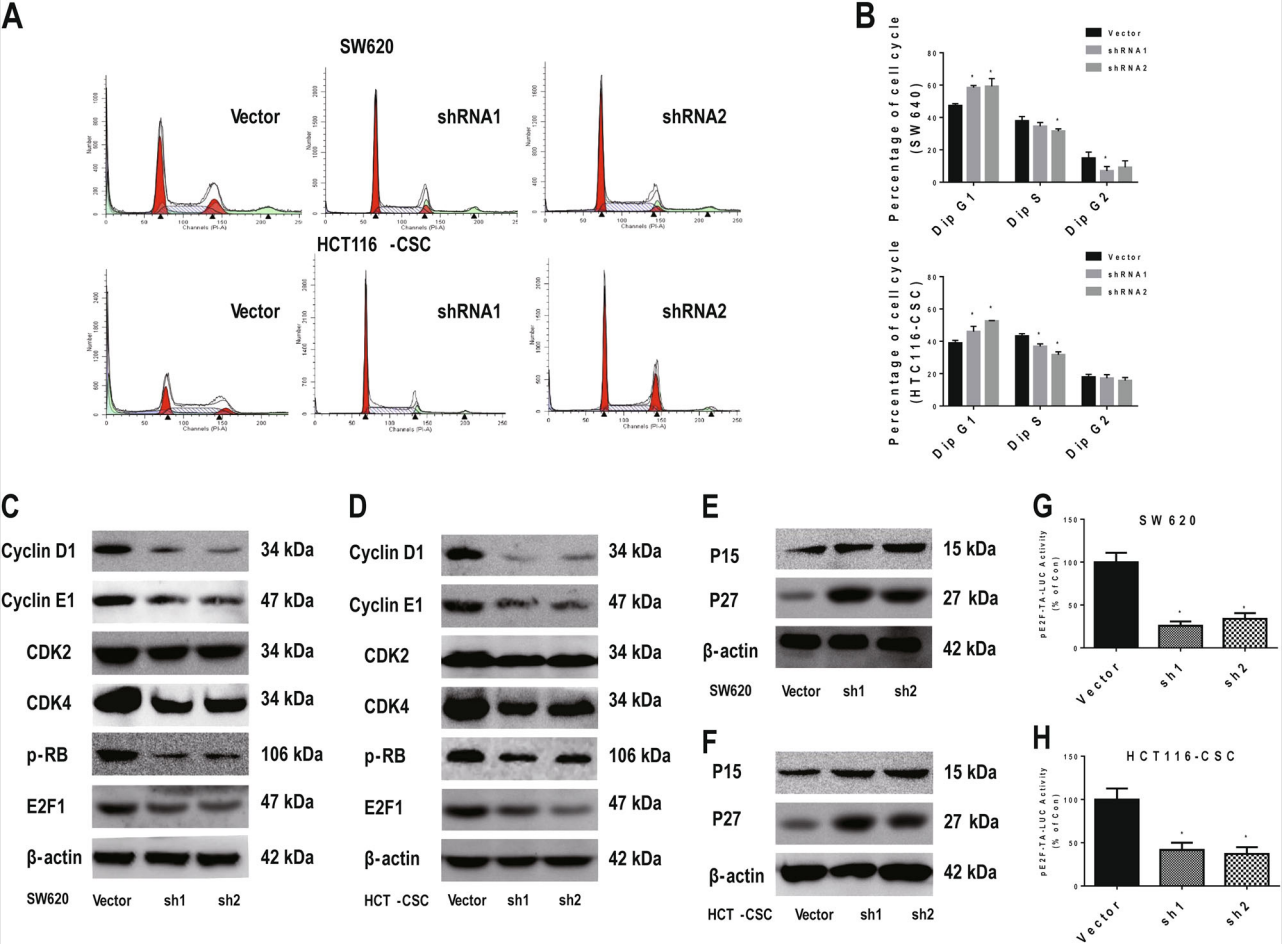
FIBP knockdown inhibited the G1–S-phase transition in CRC cells. **a** Representative cell cycle data for SW620 cells and HCT116-CSCs with/without FIBP knockdown. **b** Summary of the quantitative cell cycle analysis results in SW620 cells and HCT116-CSCs with/without FIBP knockdown. **c**, **d** Western blot results showing that the cyclin D1, CDK4, p-RB, and E2F1 levels were significantly decreased after FIBP knockdown in SW620 cells (**c**) and HCT116-CSCs (**d**). **e**, **f** The P15 and P27 levels were significantly increased after FIBP knockdown in SW620 cells (**e**) and HCT116-CSCs (**f**). **g**, **h** The luciferase activity driven by the E2F motif was significantly decreased after FIBP knockdown in SW620 cells (**g**) and HCT116-CSCs (**h**). *p < 0.05 compared with the Vector control group

To test whether FIBP controls cell proliferation through cell cycle regulation, we performed a cell cycle analysis via flow cytometry. Knockdown of FIBP significantly increased the percentage of cells in G1 phase and decreased that of cells in S phase for both SW620 cells and HCT116-CSCs (Fig. 3a, b). We then focused on exploring the molecular mechanisms through which FIBP promoted G1 to S transition by evaluating the expression of checkpoint proteins for G1–S-phase progression. While decreased levels of cyclin D1, cyclin E2, CDK4, p-RB, and E2F1 were observed in both SW620 cells and HCT116-CSCs with reduced FIBP expression (Fig. 3c, d), the levels of P15 and P27 were significantly increased (Fig. 3e, f). However, the level of CDK2 remained largely unchanged upon silencing of FIBP. Moreover, dual luciferase reporter assays showed that FIBP knockdown decreased the transcription activity driven by the E2F motif in both SW620 cells (Fig. 3g) and HCT116-CSCs (Fig. 3h). Taken together, our results suggest that FIBP promotes G1–S-phase transition and sustains cell cycle signaling for G1–S-phase progression.

### FIBP knockdown enhanced chemotherapy-induced apoptosis and attenuated stem markers' expression in HCT116-CSCs

**Fig. 4:**
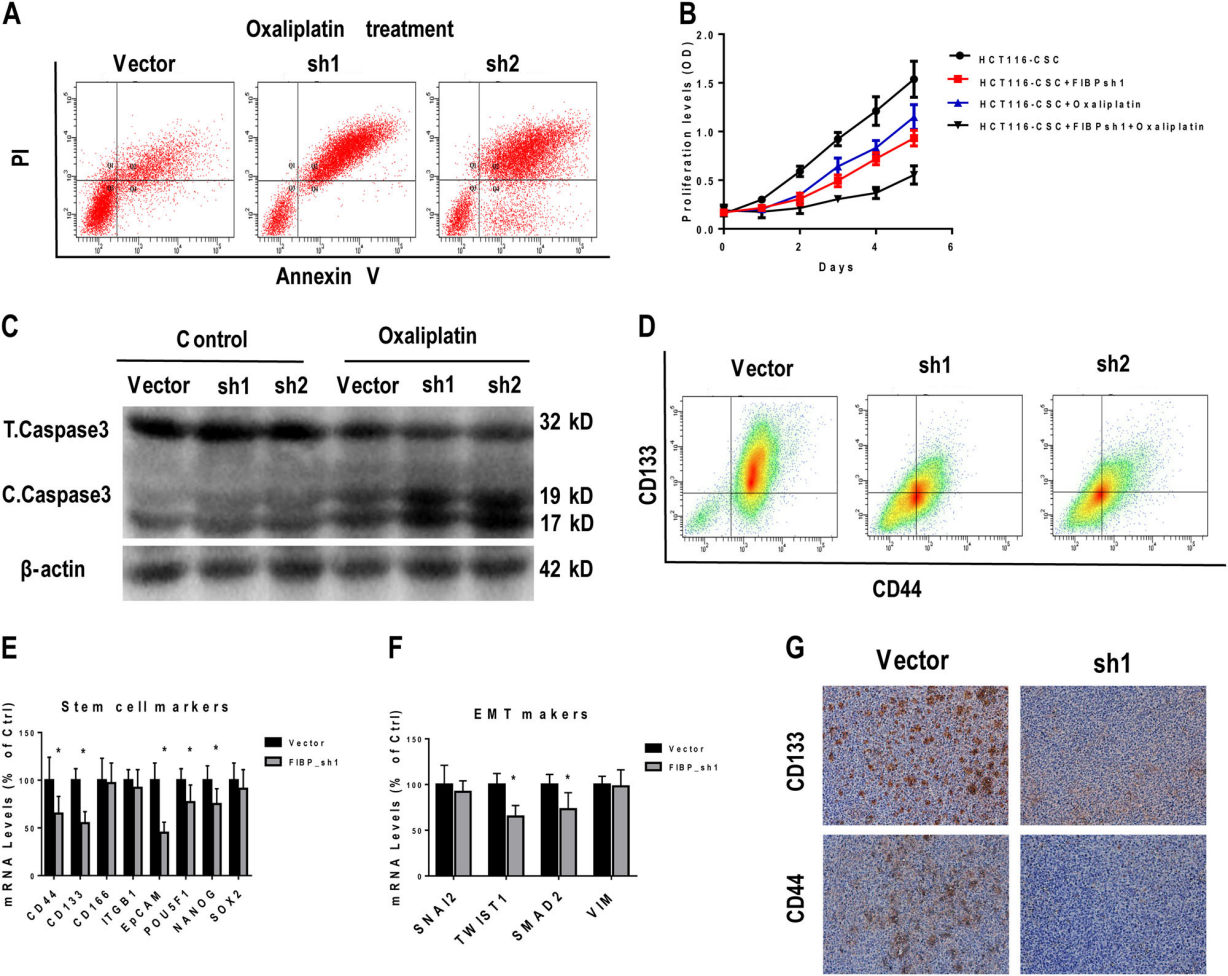
FIBP knockdown enhance the chemotherapy effect and attenuated stemness markers in HCT116-CSCs. **a** Representative flow cytometric profile of cell apoptosis analysis of HCT116-CSCs with/without FIBP knockdown in the presence of 100 μg/ml oxaliplatin during culture. **b** Proliferation curves of HCT116-CSCs with or without FIBP knockdown in the presence/absence of 100 μg/ml oxaliplatin during culture. **c** Western blot results of cleaved/total caspase 3 for HCT116-CSCs with or without FIBP knockdown in the presence/absence of 100 μg/ml oxaliplatin during culture. **d** The expression levels of CD133 and CD44 in HCT116-CSCs with or without FIBP knockdown measured by flow cytometry. **e**, **f** The expression levels of stem cell markers (**e**) and EMT markers (**f**) in HCT116-CSCs. **g** Representative immunochemistry staining results of xenograft tumors from nude mice inoculated with HCT116-CSCs pre-infected with control or FIBP-shRNA lentivirus (magnification, ×200). *p < 0.05 compared with the control Vector group

Oxaliplatin, sold under the brand name Eloxatin, is frequently used to treat CRC^16^. To investigate the effect of FIBP knockdown on CR, we first evaluated HCT116-CSC apoptosis and proliferation in the presence of 100 μg/ml oxaliplatin during in vitro culture. Remarkably, combined administration of FIBP short hairpin RNA (shRNA) and oxaliplatin synergistically inhibited cell proliferation and maybe partly by inducing cell apoptosis (Fig. 4a, b). Moreover, similar results were obtained when HCT116-CSCs were treated with other chemotherapy drugs, namely, 5-fluorouracil (5-FU) (Figure S5A and B) and Diamminedichloroplatinum (DDP) (Figure S5C and D). The slowed proliferation was also supported by the fact that cleaved Caspase 3 was significantly increased after FIBP knockdown in HCT116-CSCs with oxaliplatin treatment (Fig. 4c). On the other hand, silencing of FIBP profoundly attenuated the expression of the CSC markers CD44 and CD133 (Fig. 4d). To further investigate whether FIBP promotes stemness of CSCs, we quantitated the expression of eight stem cell markers and four epithelial–mesenchymal transition (EMT) markers via quantitative reverse transcriptase–PCR (qRT-PCR). FIBP silencing markedly reduced the expression of the stem cell markers CD44, CD133, EpCAM, POU5F1, and NANOG (Fig. 4e) as well as that of the EMT markers TWIST1 and SMAD2 (Fig. 4f), in HCT116-CSCs. Furthermore, tumors from mice inoculated with FIBP-silenced HCT116-CSCs also displayed decreased CD133 and CD44 expression (Fig. 4g). Collectively, our results show that FIBP enhances chemotherapy-induced apoptosis and attenuates stemness in CRC cells.

### Cyclin D1 is a key downstream target of FIBP in chemoresistant CRC cells

**Fig. 5:**
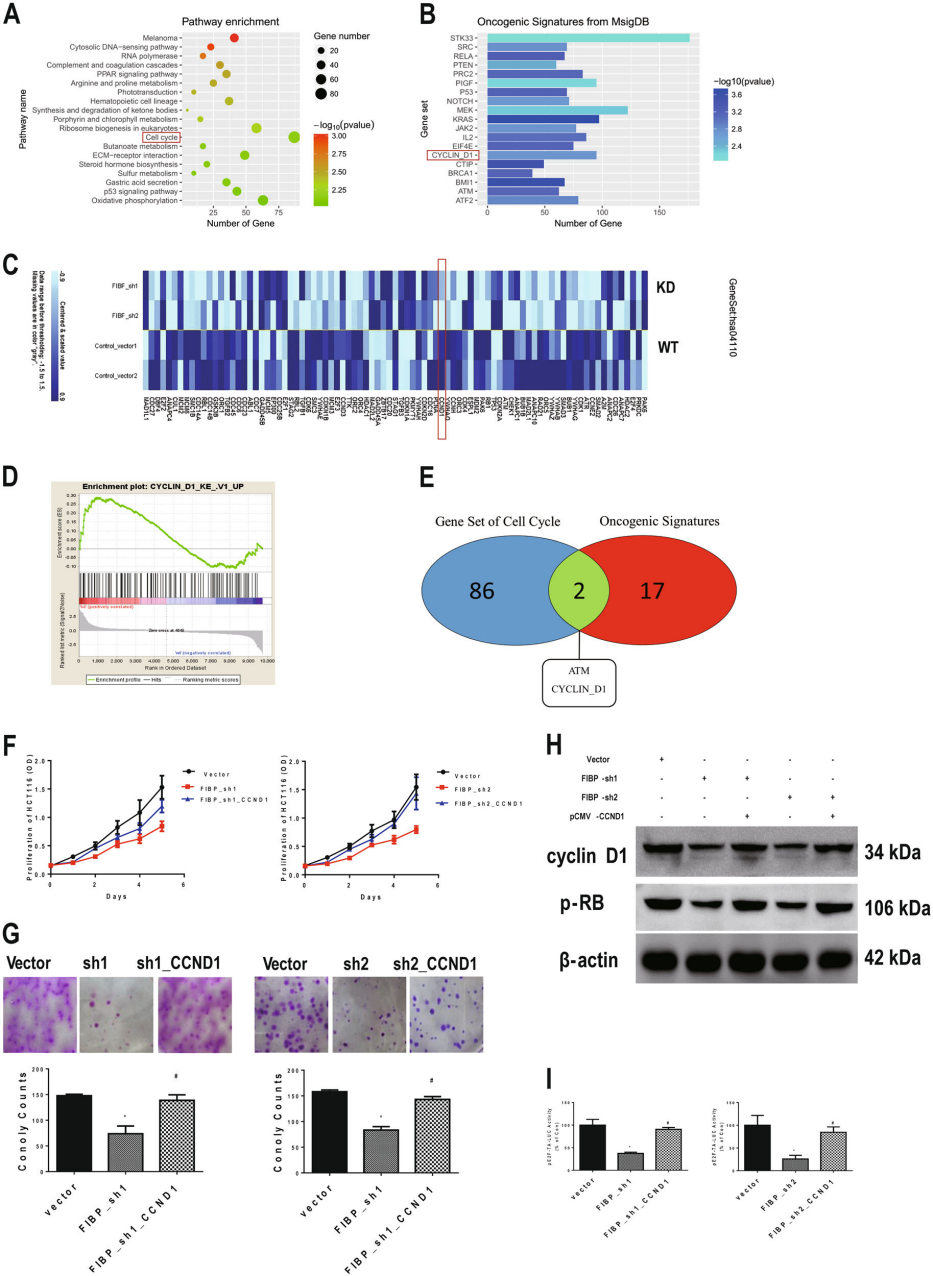
Cyclin D1 is a key downstream target of FIBP and participates in FIBP-regulated CRC cell proliferation. **a** KEGG pathway enrichment analysis of genes in HCT116-CSCs with/without FIBP knockdown. The representative pathways include cell cycle-associated genes. **b** Heatmap showing the expression levels of molecules in the cell cycle gene set. **c** Oncogenic gene signatures enrichment analysis in HCT116-CSCs with/without FIBP knockdown. **d** Cyclin D1 is simultaneously present in gene sets involving both cell cycle regulation and oncogenic signatures. **e** Gene set enrichment analysis (GSEA) of gene signatures of cyclin D1-regulated genes in HCT116-CSCs with/without FIBP knockdown. **f**, **g** Comparisons of the proliferation curves (**f**) and colony-formation abilities (**g**) in control HCT116-CSCs, HCT116-CSCs with FIBP knockdown alone, and HCT116-CSCs with FIBP knockdown and restored cyclin D1 expression via CCND1 plasmid transfection. **h** Western blotting results showing the expression levels of cyclin D1 and p-RB in HCT116-CSCs with/without FIBP knockdown and cyclin D1 re-expression. **i** Luciferase activity driven by the E2F motif increased following restoration of cyclin D1 expression in HCT116-CSCs with silenced FIBP. *p < 0.05 compared with the Vector control group. ^#^*p* < 0.05 compared with the FIBP-shRNA alone group

To characterize gene expression changes associated with FIBP expression, we performed RNA-seq analysis using mRNA isolated from HCT116-CSCs with and without FIBP knockdown. Pathway set enrichment from the KEGG database showed that 86 pathways passed the filtering criteria (*p* < 0.01, a representative pathway is shown in Fig. 5a). Genes involved in cell cycle process were significantly differentially expressed in cells with FIBP knockdown (*p* = 0.007), which is consistent with our previous protein quantification results. Specifically, 41 of the 87 genes showed statistically significant changes between control cells and FIBP-silenced HCT116-CSCs (*p* < 0.05, Fig. 5b). To determine the key downstream target of FIBP, we performed gene set enrichment analysis (GSEA) for Oncogenic Signatures in MsigDB, and a total of 57 sets, including 19 genes, passed the filtering criteria (*p* < 0.01, Fig. 5c). These genes were considered to be potential downstream targets of FIBP. A Venn diagram showed that CCND1 (encoding cyclin D1) was simultaneously present in both the cell cycle and oncogenic signatures sets (Fig. 5d). The GSEA plot also indicated a significant upregulation of CCND1 signatures in HCT116-CSCs with FIBP knockdown (false discovery rate (FDR) *q*-value = 0.005; Fig. 5e). Next, we used pCMV-CCND1 transfection to restore cyclin D1 expression to assess whether it is a downstream target of FIBP. As expected, ectopic expression of cyclin D1 abrogated the proliferation inhibition that resulted from FIBP knockdown (*p* < 0.001, Fig. 5f) and significantly reversed the decrease in HCT116-CSC colony number (*p* < 0.001, Fig. 5g). In addition, supplementation of cyclin D1 restored Rb phosphorylation (Fig. 5h) and the transcriptional activity of the E2F motif (Fig. 5i) in FIBP-silenced HCT116-CSCs. Thus our results suggest a positive correlation between FIBP and cyclin D1 in controlling the proliferation of HCT116-CSCs.

### FIBP inactivated glycogen synthase kinase 3β (GSK3β) signaling by binding GSK3β and inhibiting its phosphorylation at Tyr216

**Fig. 6:**
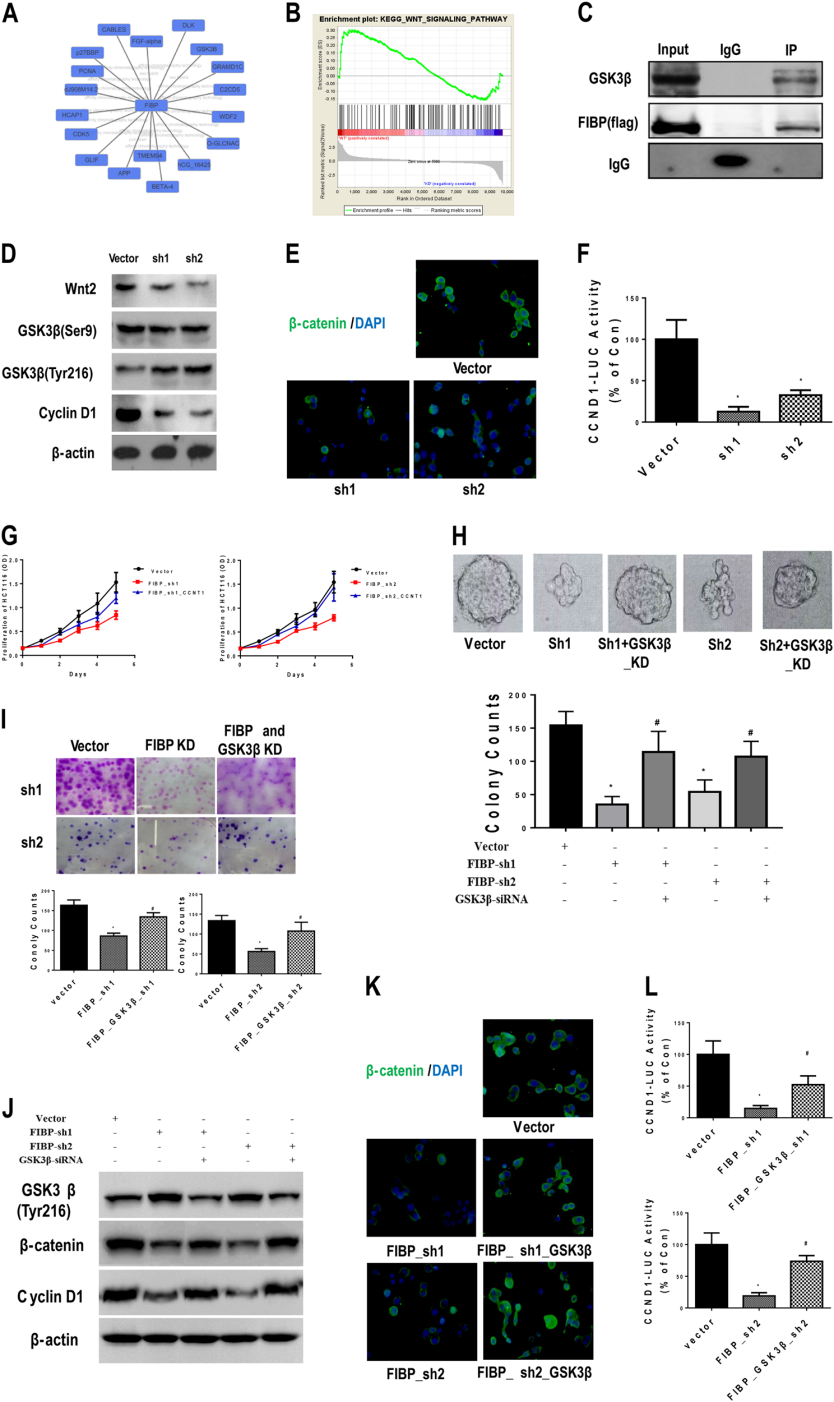
FIBP inhibited the phosphorylation of GSK3β ^Tyr216^ and activated β-catenin/TCF signaling. **a** Speculated protein interactions for FIBP, analyzed from four databases (HPRD, BioGRID, IntAct, and MINT). **b** GSEA of the Wnt signaling pathway in HCT116-CSCs with/without FIBP knockdown. **c** Co-immunoprecipitation analysis showing the interaction between Flag-tagged FIBP (Flag-FIBP) and GSK3β. **d** Western blotting results showing the levels of Wnt2, GSK3β (Ser9 and Tyr216), and cyclin D1 in HCT116-CSCs with or without FIBP knockdown. **e** Representative immunofluorescence staining results showing β-catenin (green) and DAPI (blue) in HCT116-CSCs with or without FIBP knockdown (magnification, ×400). **f** Luciferase activity driven by the cyclin D1 promoter was decreased in HCT116-CSCs with FIBP knockdown. **g**–**i** The proliferation curves (**g**), sphere-formation abilities (**h**), and colony-formation abilities (**i**) of control HCT116-CSCs, HCT116-CSCs with FIBP knockdown alone, and HCT116-CSCs with combined knockdown of FIBP and GSK3β. **j** Western blotting results showing the levels of phosphorylated GSK3β ^Tyr216^, β-catenin, and cyclin D1 in HCT116-CSCs with FIBP knockdown alone or combined knockdown of FIBP and GSK3β. **k** Immunofluorescence staining of β-catenin (green) and DAPI (blue) staining in HCT116-CSCs with FIBP knockdown alone or combined knockdown of FIBP and GSK3β (magnification, ×400). **l** Luciferase activity driven by the cyclin D1 promoter increased following reversal of the GSK3β levels in HCT116-CSCs with FIBP knockdown. *p < 0.05 compared with the Vector control group. ^#^*p* < 0.05 compared with the FIBP-shRNA group

To uncover the molecular mechanisms underlying the connection between FIBP and cyclin D1, we searched four protein–protein interactions databases (HPRD, BioGRID, IntAct, and MINT). We identified 18 FIBP-interacting proteins (Fig. 6a), among which GSK3β was selected for further study because it is widely recognized as a protein upstream of cyclin D1 and is closely related to tumor development. This was supported by the GSEA plot from the KEGG database that indicated that the Wnt pathway might be involved in FIBP-induced biological processes (FDR *q*-value = 0.011, Fig. 6b). Subsequent immunoprecipitation experiments confirmed the interaction between FIBP and GSK3β (Fig. 6c). Moreover, silencing of FIBP significantly decreased the expression of Wnt2 and cyclin D1 but substantially increased the phosphorylation of GSK3β at Tyr216. However, the levels of GSK3β ^Ser9^ phosphorylation were nearly unchanged (Fig. 6d). The regulation of GSK3β signaling by FIBP was also evidenced by the drastically decreased β-catenin levels revealed by immunofluorescence staining of HCT116-CSCs with FIBP knockdown (Fig. 6e), as well as by the decreased luciferase activity driven by the T-cell factor (TCF) motif in the CCND1 promoter (−500/0) (Fig. 6f). Taken together, our data demonstrate that FIBP binds to GSK3β and inhibits GSK3β phosphorylation at Tyr216 and suggest that FIBP might activate β-catenin/TCF/cyclin D1 signaling.

### GSK3β knockdown restored the proliferation ability of HCT116-CSCs with silenced FIBP

To determine the role of GSK3β in FIBP silencing-induced inhibition of cell proliferation, we used small interfering RNA (siRNA) transfection to knockdown GSK3β. Interestingly, GSK3β knockdown alone led to significantly decreased proliferation in both SW620 cells and HCT116-CSCs (Figure S6). However, administration of siRNA against GSK3β reversed the slowed growth of HCT116-CSCs with FIBP knockdown (Fig. 6g). The defective proliferation ability caused by reduced FIBP expression in HCT116-CSCs was also restored by GSK3β knockdown, as evidenced by both the colony-forming assay (Fig. 6i) and sphere-formation assay (Fig. 6h) results. Additionally, silencing of GSK3β reduced the level of phosphorylated GSK3β ^Tyr216^ and restored the expression of β-catenin and cyclin D1 (Fig. 6j). Consistent with the western blot assays, our immunofluorescence results demonstrated that β-catenin had comparable expression in control cells and HCT116-CSCs with silencing of both FIBP and GSK3β (Fig. 6k). Furthermore, GSK3β knockdown reversed the decrease in luciferase activity driven by the CCND1 promoter in HCT116-CSCs with silenced FIBP (Fig. 6l). Collectively, our data imply that the GSK3β/β-catenin pathway plays a key role in FIBP-regulated cyclin D1 expression and CRC development. Interestingly, overexpression of β-catenin only restored proliferation in HCT116-CSCs with FIBP knockdown (Figure S7A and B) but was not able to reverse the expression patterns of stemness markers (Figure S7C and D). These findings suggest that the expression of stemness markers might be independent of GSK3β/β-catenin signaling.

### GSK3β knockdown restored FIBP-regulated chemotherapy effects and stemness markers via DNA demethylation

**Fig. 7:**
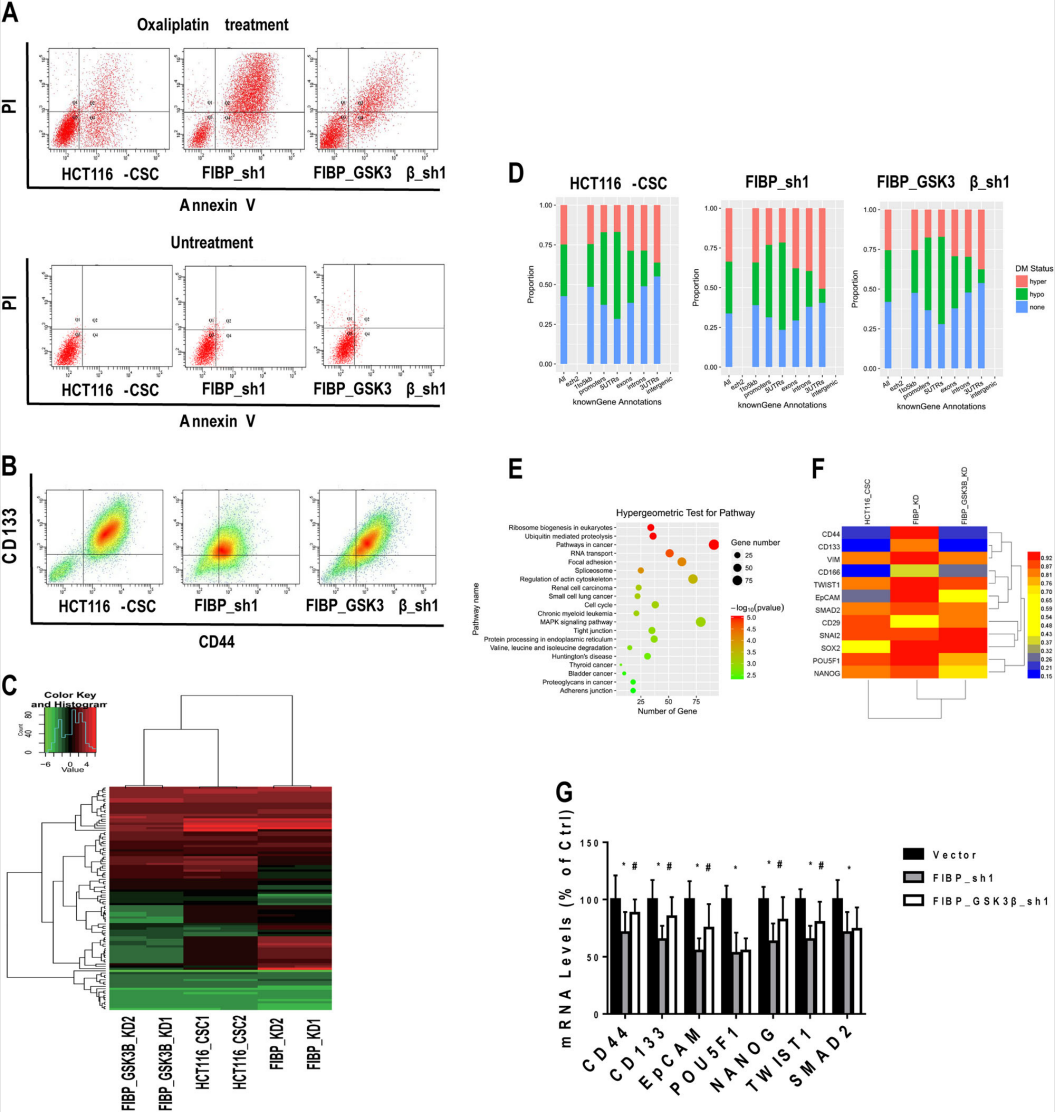
GSK3β knockdown restored the FIBP silencing-induced reduction in stemness markers of HCT116-CSCs via DNA demethylation. **a**, **b** Representative flow cytometric profile showing cell apoptosis (**a**) and surface CD44/CD133 expression (**b**) in control HCT116-CSCs, HCT116-CSCs with FIBP knockdown alone, and HCT116-CSCs with combined knockdown of FIBP and GSK3β in the presence of 100 μg/ml oxaliplatin during culture. **c** Heatmap of beta values for top 200 differentially methylated sites in control HCT116-CSCs, HCT116-CSCs with FIBP knockdown alone, and HCT116-CSCs with combined knockdown of FIBP and GSK3β. **d** Gene annotations with the proportions of differentially methylated regions in each group shown in **a**, **b**. **e** Hypergeometric test for the top 1000 genes with differentially methylated regions based on the KEGG database. **f** Heatmap of beta values for gene regions of stem cell markers and EMT markers. **g** The expression of stem cell markers and EMT markers in control HCT116-CSCs, HCT116-CSCs with FIBP knockdown alone, and HCT116-CSCs with combined knockdown of FIBP and GSK3β. *n* = 8 for each group. *p < 0.05 compared with the Vector control group. ^#^*p* < 0.05 compared with the FIBP-shRNA group

To further explore how FIBP influences stemness and determine whether GSK3β is involved in this process, we measured the oxaliplatin-induced apoptosis and CD44/CD133 levels via flow cytometry. Silencing of GSK3β drastically reversed FIBP knockdown-induced cell apoptosis with oxaliplatin treatment (Fig. 7a) and reduction in CD44/CD133 expression (Fig. 7b) in HCT116-CSCs. Given that the stemness process is closely related to DNA methylation patterns^17,18^ and GSK3β is able to regulate DNA methylation^19,20^, we detected the levels of 5-methyl-deoxycytidine (5-mdC) in cells with altered FIBP/GSK3β expression using high-performance liquid chromatography. Notably, 5-mdC levels were increased after FIBP knockdown and were reversed by GSK3β knockdown but not by overexpression of β-catenin (Figure S8). This implies that the action of methylation might be downstream of GSK3β but upstream of β-catenin. Next, we determined the genome-wide methylation pattern in HCT116-CSCs using an Illumina HumanMethylation450 BeadChip. Among the 485,577 assays for CpG sites, the methylation status in 10,539 promoter sites, involving 3923 genes, was significantly changed among the three groups (*p* < 0.01). A heatmap of beta values and cluster analysis show that the DNA methylation patterns were similar in HCT116-CSCs and HCT116-CSCs with FIBP/GSK3β knockdown (Fig. 7c). After counting annotated and methylation sites, we found that the number of sites with hypermethylation increased in HCT-CSCs with FIBP knockdown (23.87% vs. 32.12%), and this pattern was reversed after additional GSK3β knockdown (32.12% vs. 26.62%) (Fig. 7d). Our hypergeometric test for the top 1000 genes with differential methylation demonstrated that a series of tumorigenicity-related signals were involved in the FIBP-regulated methylation patterns. Notably, the KEGG pathway set “proteoglycans in cancer” contained tumor stemness processes indicated by CD44 (Fig. 7e). In addition, the methylation status of 12 genes that are either stemness markers or EMT markers was checked. Remarkably, the gene regions of stemness markers (CD44, CD133, CD166, EpCAM, POU5F1, and NANOG) and EMT markers (VIM, TWIST1, and SMAD) were methylated in cells with FIBP knockdown and were partially demethylated after additional GSK3β knockdown (Fig. 7f and Table S3). Subsequent qRT-PCR analysis confirmed that the expression of five downregulated genes (CD44, CD133, EpCAM, NANOG, and TWIST1) in HCT116-CSCs with silenced FIBP were partially restored by GSK3β knockdown (Fig. 7g) or treatment with a DNA methyltransferase inhibitor (5-aza-2′-deoxycytidine) (Figure S8C). In summary, our results revealed that FIBP may promote CRC cell proliferation and stemness via inhibition of the GSK3β-mediated β-catenin/cyclin D1 pathway and methylation of stemness genes (Figure S9).

## Discussion

In this study, we found that FIBP knockdown inhibited the growth and stem makers' expression of CRC cells with CR via GSK3β-related signaling. Mechanistically, we showed that FIBP bound to GSK3β, which inhibited phosphorylation of GSK3β at Tyr216 and activated β-catenin/TCF signaling to accelerate cell cycle and promote tumor growth. In addition, FIBP knockdown sensitized chemoresistant cells and attenuated stemness, which involved methylation of stemness genes by factors downstream of GSK3β but upstream of β-catenin. To the best of our knowledge, this is the first report describing the role of FIBP in CRC with CR, and our results provide insight into how FIBP regulates GSK3β-related pathways. Importantly, we show that FIBP plays an important role in the tumorigenic potential of CSCs, illuminating the potential of FIBP as a new therapeutic target for chemoresistant CRC.

There is increasing evidence showing that chemical resistance is attributable to domination of CSCs^21,22^. Currently, highly tumorigenic and self-renewing colorectal CSC populations in human colon cancers have been successfully enriched and identified with a series of markers, including CD133, CD44, and CD107^7,23^. CRC cells with these markers remained undifferentiated, display a much more proliferative phenotype, and are associated with a poor prognosis^7^. Inactivation of tumor-suppressor genes and excessive proliferative signaling are considered to be the causes of CSC self-renewal capability. For example, p53 and its regulatory pathway (Np73, SIRT1, etc.) are regarded as therapeutic targets for CSCs because restoration of p53 function induced cell death^24,25^. Excessive proliferation signaling pathways, such as activated phosphatidylinositol 3-kinase/AKT, mitogen-activated protein kinase (extracellular signal–regulated kinase), GSK3β/Wnt, and epidermal growth factor signaling, were also confirmed to be vital for colon CSC tumorigenicity^26,27^, which is always accompanied by high expression of proliferation-related genes.

FIBP is an intracellular protein that binds selectively to aFGF, which participates in cell proliferation by stimulating mitogenesis^28^. This suggests that FIBP might be involved in mitogenic activity and cell proliferation. A previous study reported that FIBP-depleted breast cancer cells displayed impaired proliferation and decreased migration^29^. Based on a published dataset (GSE9328), we also found that FIBP was highly expressed in skin carcinogenesis^30^. More importantly, FIBP was found to be expressed at higher levels in saracatinib-resistant CRC than in saracatinib-sensitive cancer based on further in-depth analysis of Gene Expression Omnibus (GEO) datasets (GSE36006)^31^. Although these data show that FIBP might be correlated with carcinogenesis and drug resistance, the functions of FIBP in specific aspects of tumorigenesis remain largely unknown. For successful long-term CRC therapy, it is critical to target both CSCs and non-CSC tumor cells^25^. Our results indicate that FIBP expression is positively correlated with poor prognosis in CRC patients, and knockdown of FIBP inhibits the proliferation of CRC cells. Thus our data further strengthen the potential of FIBP as a target for successful long-term CRC therapy. A few previous studies have reported that FIBP is involved in tumor cell cycle processes. For example, FIBP can bind to KIAA0528 and CDK5 to form a stable complex, which participates in breast cancer cell growth and migration^29^. Our current study establishes the involvement of FIBP in cell cycle modulation in CRC cells through regulation of the key downstream target cyclin D1, which further supplements the underlying molecular mechanisms of the role of FIBP in tumorigenesis.

Hyperactivation of the Wnt/β-catenin signaling pathway has been found to be associated with various types of human cancers, most notably CRC. Numerous reports have highlighted the importance of Wnt/β-catenin signaling in CSC self-renewal and oncogenesis^32^. Owing to APC and CTNNB1 mutations, β-catenin signaling is hyperactivated, and several downstream targets of β-catenin, such as c-myc, cyclin D1, and PPARdelta, have been identified as markers of tumor development^33,34^. Hyperactivated Wnt/β-catenin signaling has also been shown to be an important characteristic of CSCs in human CRC^35^. For example, Gastrin, a powerful self-renewal promoter in cancer, is a functionally relevant downstream target of the β-catenin signaling pathway and can further enhance Wnt pathway signaling^36,37^. Therefore, targeting the Wnt pathway or its regulatory signaling is considered a potentially effective approach for treating CRC^37,38^. GSK3β is a serine–threonine kinase that can form a destruction complex with Axin, APC, PP2A, and CK1α. After phosphorylation of GSK3β at Tyr216, this complex degrades β-catenin by targeting it for ubiquitination and therefore inhibits the Wnt/β-catenin pathway. GSK3β plays an important but paradoxical role in cancer processes. On one hand, GSK3β can inhibit β-catenin and subsequent tumor progression, and its downstream targets (c-myc, cyclin D1, PPARdelta) are also reported to be involved in this process^39–41^. On the other hand, GSK3β also modulates CSC stemness and cancer cell migration through Wnt-independent mechanisms, such as regulation of histone methylation and other feedback signaling^42,43^. Furthermore, GSK3β has been reported to regulate the resistance to chemotherapy in a variety of cancer cells. For instance, increased phosphorylation of GSK3β (Ser9) is observed in cisplatin-resistant ovarian cancer cells^44^, while inhibition of GSK3β activity contributes to drug resistance in breast cancer cells^45^ and neuroblastoma cells^46^.

Consistent with these reports, our data show that knockdown of FIBP in HCT116-CSCs sensitized chemoresistant cells and enhanced the phosphorylation of GSK3β at Tyr216 but not Ser9. Our results underscore the importance of the GSK3β/β-catenin/cyclin D1 axis in FIBP silencing-induced therapeutic effects. However, in some cases, inhibition of GSK3β activity also inhibits tumor growth and sensitizes cells to chemotherapy^47^. We also found that direct knockdown of GSK3β inhibited the proliferation of SW620 cells and HCT116-CSCs but not HCT116-CSCs with silenced FIBP. Thus it is plausible that the interaction between FIBP and GSK3β but not the expression level of GSK3β predominantly matters in determining cell proliferation. In our study, cells with higher FIBP expression exhibited accumulation of β-catenin, which contributed to CRC proliferation. Hence, it is possible that restoring GSK3β to normal levels may help inhibit β-catenin accumulation and subsequently block cell cycle progression. However, in cells with overexpression of GSK3β, an oncogenic pathway independent of β-catenin may become dominant and promote tumor development. Therefore, further studies are warranted to comprehensively investigate the precise relationship between GSK3β-related signaling and tumorigenesis in different CRC cell lines. Moreover, it has been reported that DNA methylation patterns are associated with the clonal expansion and cancer stem cell dynamics in CRC^17,18^. Hypomethylation of stem markers was found in the tumorigenesis process and promoted drug resistance^48,49^, However, the mechanisms still remain largely unknown. GSK3β has been identified as a fundamental player in regulating DNA methylation of imprinted loci in stem cells^19,20^. Our study revealed that FIBP regulated CRC cell stemness via direct methylation of stemness marker genes, which was dependent on GSK3β signaling, further underscoring the critical role of DNA methylation in stem cell self-renewal and stemness.

In summary, our findings reveal an important role of FIBP in modulating CSC stemness and CR through the GSK3β-related β-catenin/cyclin D1 axis and DNA methylation activity in CRC cells. FIBP may serve as a novel therapeutic target for chemoresistant CRC through inhibition of GSK3β-related pathways, and this may lead to novel therapeutic strategies against drug-resistant CRC.

## Methods

### Cell culture and chemoresistant cell lines established

Human CRC cell lines (LoVo, SW48, LS180, SW620, HCT116, and SW480) and normal human colonic epithelial cell lines (NCM460 and FHC) were purchased from the Cell Bank of the Chinese Academy of Sciences (Shanghai, China). Cells were cultured in RPMI1640 medium (Gibco, New York, USA) supplemented with 10% fetal bovine serum (FBS) (Gibco BRL), 100 units/ml penicillin, and 100 μg/ml streptomycin in a humidified atmosphere containing 5% CO_2_ at 37 ℃.

Oxaliplatin-resistant HCT116 cell lines were established based on previous experience and further optimization. Briefly, 20 ng/ml L-OHP was initially used to induce drug resistance in HCT-116 parental cells, and thereafter, the concentration of L-OHP was gradually increased during the long cycle of drug resistance selection. Cells with CSC markers detected with an ALDEFLUOR™ Kit (STEMCELL Technologies, USA) were sorted by flow cytometry. The workflow and fluorescence-activated cell sorter gating strategy are shown in Figure S1A. Sorted HCT-116 cells were seeded in medium containing 5 mg/ml L-OHP to maintain the drug-resistant phenotype. The established HCT116 cell line (HCT116-CSC) was positive for two CSC markers CD44 and CD133 and validated for their resistance to chemotherapeutic drugs, including oxaliplatin, 5-FU, and cisplatin (Figure S1C–E).

### Lentivirus infection and plasmid/siRNA transfection

Stable FIBP knockdown cell lines were produced by infection with lentivirus containing shRNA against FIBP, and restoration of cyclin D1, GSK3β and β-catenin expression was achieved by cell transfection with specific plasmids. The lentivirus vector encoding shRNA targeting FIBP was synthesized by Genepharma (Shanghai, China). Target cells (SW1116, SW620, and HCT116 cell lines) were infected with pCDH-shRNA-FIBP or pCDH-shRNA-NC lentivirus in the presence of 5 μg/ml polybrene. The stable cell lines were established after 1 week of selection with puromycin (1 μg/ml), and knockdown of FIBP protein was subsequently validated by western blotting.

CCND1 gene (NM_053056.2, encoding cyclin D1) and CTNNB1 gene (NM_001098209, encoding β-catenin) cDNAs were synthesized by Genepharma (Shanghai, China), amplified, and cloned into a pCMV vector. siRNA oligos for GSK3β were synthesized by Genepharma (Shanghai, China). Cells were transfected with plasmids or siRNA using Lipofectamine 2000 (Life Technologies, USA) according to the manufacturer’s instructions. Cells were collected for western blotting to validate the silencing of targeted genes 24 h after transfection. The sequences of all the shRNA and primer oligos used in this study are shown in Table S1.

### Cell proliferation, colony formation, and cell migration assays

Cell proliferation was measured via CCK-8 reagent (Cell Counting Kit-8, Dojindo Kumamoto, Japan) following the manufacturer’s instructions. Briefly, cells were seeded in 24-well plates (1 × 10^4^ cells/well) and cultured for the indicated time points. Then 20 μl of CCK-8 reagent was added into each well, and 3 h later, the absorbance at 450 nm was measured using a microplate reader (S5 Versa Analyzer, USA). For colony-formation assay, cells were seeded in a 6-well plate (500 cells/well) and cultured for 2 weeks. After fixation with methanol for 5 min, the cells were stained with 0.1% crystal violet. Colonies containing >50 cells were counted for statistical analysis. For transwell migration assays, transwell chambers equipped with 8-μm membranes (Life Technologies, USA) were used. Briefly, cells were grown in serum-free culture medium overnight. After trypsinization, cells were resuspended at a density of 1.0 × 10^6^/ml in serum-free culture medium and then plated in the upper chambers (200 μl). Meanwhile, the lower chambers were filled with 600 μl of medium containing 10% FBS. After 24 h, the cells were fixed with formaldehyde for 5 min and then stained with 0.1% crystal violet. Non-migratory cells in the chambers were wiped away, and the membranes were removed. Cells in the lower chambers were mounted onto glass slides and counted. For all the assays, the cells in each group were plated in three replicate wells, and each experiment was performed with triplicate technical repeats.

### Tumor xenografts

BALB/c nude mice, 5–6 weeks old, were purchased from the Experimental Animal Center of Southern Medical University and maintained under standard pathogen-free conditions. Tumor xenografts were established as described previously by subcutaneously injecting 1 × 10^7^ cells into the right flank of nude mice (5 mice per group, randomly assigned, not a blinded method). Tumor growth was measured using calipers from day 7 to day 20 according to the following formula: *LWH*π/6, where *L* is the length, *W* is the width, and *H* is the height of the tumor. All animal studies (including the mouse euthanasia procedure) were performed in compliance with the regulations and guidelines of the Institutional Animal Care and Use Committees (IACUC) of Southern Medical University and the Association for Assessment and Accreditation of Laboratory Animal Care International (AAALAC).

### Immunohistochemistry (IHC) and microarray slides

Tissue samples were dissected, fixed in 4% paraformaldehyde, and sliced into 4-mm paraffin-embedded pieces. After dewaxing, nonspecific peroxidase activity was blocked with 3% H_2_O_2_ for 15 min, followed by three 5-min washes with phosphate-buffered saline (PBS). Sections were then incubated in 5% bovine serum albumin (BSA)–PBS for 30 min and probed with the selected primary antibodies (anti-FIBP, 1:100 and anti-Ki-67, 1:250; Abcam) at 4 °C overnight. Immunostaining with horseradish peroxidase (HRP)-conjugated secondary antibodies was then performed for 4 h at room temperature (RT). Human CRC tissue microarray slides (Cat: HCol-Ade180Sur-07) were supplied by Shanghai OUTDO Biotech (Shanghai, China). A total of 89 CRC samples with adjacent normal colon tissues were obtained along with detailed patient information, including age, gender, metastasis status, pathological grade, tumor size, TNM stage, and AJCC stage. The positive staining intensity of tumor cells was scored as follows: negative (0), weak (1), moderate (2) and strong (3). The percentage of positively stained cells was divided into four categories: <25% (1), 25–50% (2), 51–75% (3), and >75% (4). The final staining scores were calculated as the intensity × the staining percentage to achieve a score between 0 and 12. A final score >6 was defined as high expression and ≤6 was defined as low FIBP expression. Kaplan–Meier analysis and Cox regression were used to determine the survival statistics of patients. All patients provided written informed consent to allow the tissue staining and the qRT-PCR analysis of genetic data. The tumor specimens were obtained from a nested cohort study of the patients with CRC. Briefly, tumor specimens were obtained by colonoscopy prior to chemotherapy. The effects of chemotherapy on the tumor were evaluated by the Response Evaluation Criteria in Solid Tumors as follows: complete response (disappearance of the disease), partial response (reduction of 30%), stable disease (reduction <30% or enlargement 20%), and progressive disease (enlargement 20%). Then we classified complete response or partial response as chemosensitivity and progressive disease as CR. All the experiments involving CRC patients were approved by the Ethics Committee of the Women and Children’s Hospital Affiliated with Medical Faculty of Guangdong Medical University (no. F-2013–0091–02).

### RNA-sequencing and gene set enrichment analysis (GSEA)

Total mRNA was extracted from HCT116-CSCs with or without shRNA lentivirus infection or plasmid transfection using an RNA Extraction Kit (Qiagen K.K., Tokyo, Japan). RNA-seq was performed using an Ion Proton system for next-generation sequencing according to the manufacturer’s directions. Sequenced reads were mapped to the hg19 genome using Ion Torrent TMAP aligner with the “map4” option. The aligned RNA-seq reads against exon regions of genes were quantified with HTSeq-Count in RefSeq hg19 annotation.

GSEA was performed on mRNA expression datasets generated from HCT116-CSCs with and without FIBP knockdown, using the C2 curated gene sets and C6 oncogenic signatures gene sets (GSEA, Broad Institute) with the addition of a Wnt signature. Gene signatures were considered enriched if the FDR *q*-value < 0.05 and familywise error rate *p* value < 0.05.

### Flow cytometric analysis

Apoptosis analysis was performed using a kit from BD Biosciences, USA. Briefly, cells were incubated with annexin V-fluorescein isothiocyanate and propidium iodide for 15 min at RT. After being washed, the cells were diluted in 400 μl of annexin V-binding buffer and immediately examined using a flow cytometer. The gating was based on the control sample, and cells in both Q1 and Q3 were regarded as apoptotic cells. For CD44/CD133 analysis, cells were suspended with flow buffer solution (autoclaved PBS + 0.5% serum + 2 mM EDTA) and stained with fluorescence-labeled antibodies against CD44 and CD133 (Biolegend, San Diego, USA). Cytometric analysis was performed with the flow cytometer (Cytomics FC 500, Beckman Coulter, Indianapolis, IN, USA) and CXP software. The gate threshold is of 1 × 10^2.5^ fluorescence for each axis.

### Co-immunoprecipitation and western blotting

Cell lysates were obtained by homogenization of HCT116-CSCs with flag-tagged FIBP expression in RIPA buffer. For Flag pull-down, anti-Flag M2 affinity gel (Sigma-Aldrich) was used. For GSK3β pull-down, rabbit polyclonal anti-GSK3β (Abcam) and protein A/G PLUS-Agarose (Santa Cruz Biotechnology) were used according to the manufacturer’s manual. Protein concentration was measured using a BCA Protein Assay Kit (Thermo Fisher Scientific, Waltham, MA, USA). After boiling for 15 min, the lysates with 5× loading buffer were loaded equally on 10% sodium dodecyl sulfate polyacrylamide gels. Following electrophoresis, the proteins were transferred to polyvinylidene difluoride (PVDF) membranes at a constant 100 V for 90 min. The PVDF membranes were blocked with 5% BSA for 1 h at RT and probed with the relevant primary antibody (anti-cyclin D1, 1:800; anti-cyclin E1, 1:800; anti-CDK2, 1:1000; anti-CKD4, 1:1000; anti-p-RB, 1:500; anti-E2F1, 1:1000; anti-P15, 1:800; anti-P27, 1:800; anti-Wnt2, 1:1000; anti-GSK3β (Tyr216), 1:500, anti-GSK3β (Ser9), 1:800; anti-β-catenin, 1:1000; anti-cleaved caspase3, 1:1000; and anti-β-actin, 1:5000) at 4 °C overnight. After three washes (each 15 min) with PBS+Tween 20 (PBST), the PVDF membranes were incubated with an HRP-conjugated secondary antibody (1:10,000) for 1 h. Finally, after being washed with PBST, the blots on the membranes were visualized with enhanced chemiluminescence reagent using FUJI SUPER RX film or a CCD system (imagestation 2000 MM, Kodak, NY, USA).

### Real-time quantitative PCR (qPCR)

Total RNA from cells was extracted using the TRIzol method (Life Technologies). cDNA was synthesized using cDNA Synthesis Supermix for qPCR (cat. AT341-01; Transgen, Beijing, China) following the manufacturer’s instructions. PCR amplification was performed in a reaction volume of 20 μl containing SYBR green on a thermal cycler (Applied Biosystems 7500). The following conditions were used: 94 °C for 30 s, followed by 42 cycles of 94 °C for 5 s and 60 °C for 34 s. The data were calculated using the 2^−^^ΔΔCt^ method^50^, and the primers used are shown in Table S1.

### Immunofluorescence

Cells growing on a glass slide at approximately 80% confluence were fixed with 4% paraformaldehyde for 15 min, permeabilized with 0.25% Triton X-100 for 10 min at RT, and blocked with PBST for 30 min. After being washed with PBS, the cells were incubated with fluorescence-labeled anti-β-catenin antibody (1:200, Abcam). Cell nuclei were stained with DAPI (4’,6-diamidino-2-phenylindole, 1 μg/ml) for 10 min at RT. The fluorescence signals were observed using a Leica inverted fluorescence microscope (Leica, Germany).

### Dual luciferase reporter assay

To assay the transcriptional activity of E2F, a pE2F-TA-LUC (E2F-LUC) reporter containing multiple E2F motif constructs and the internal control reporter pGME2F-SEAP (GME2F-SEAP) were obtained from Beijing Biolab Co. (SY0198 - ZKC). HCT116-CSCs were cultured in 24-well plates (1 × 10^5^ cells/well) and co-transfected with 300 ng pE2F-TA-LUC and 10 ng pGME2F-SEAP. The cyclin D1 promoter region for TCF was located at −441 to −425 bp, and thus the promoter sequence fragment (−500 bp/0 bp) was cloned into pGL6-LUC (firefly luciferase) to generate pGL6-pcyclin D1-LUC. To verify the transcription of cyclin D1, HCT116-CSCs were co-transfected with 300 ng of pGL6-pcyclin D1-LUC and 40 ng of pRLTK (Renilla luciferase). Cells were lysed at 24 h post-transfection, and luciferase activity was measured with a Dual Luciferase Reporter Assay System (cat. E1910; Madison, WI, USA).

### Methylation profiling with a genome tiling array

Genomic DNA was bisulfite-treated and purified using an EpiTect Bisulfite Plus Kit (Qiagen K.K., Tokyo, Japan). Then 300 ng bisulfite-treated DNA was hybridized to the Illumina Infinium HumanMethylation450 BeadChip using Illumina-supplied reagents and protocols (Illumina, Inc., USA). The R package “minfi” was used to calculate the methylation level at each site as a beta value. The raw data for the genome-wide methylation study in HCT116-CSCs have been uploaded to the GEO database (GSE104271).

### Statistical analysis

Expression differences between normal colorectal tissues and CRC tissues were calculated using a chi-squared test. Kaplan–Meier analysis was used to determine the overall survival statistics, and Cox regression analysis was employed for independent correlation of individual parameters with patient overall survival. The cell proliferation and migration data were analyzed using Student’s *t* test or one-way analysis of variance (Least Significant Difference post hoc test). All the statistical analyses of the bioinformatics data shown on the heatmap were performed with the R program. A two-sided test with *p* < 0.05 was considered statistically significant.

### Accession codes

Array data are deposited at the GEO database repository under accession number GSE104271.

## Electronic supplementary material

SUPPLEMENTAL Figures 1-9

SUPPLEMENTAL Tables 1-3

## References

[1] Chen W (2016). Cancer statistics in China, 2015. CA Cancer J. Clin..

[2] Edwards BK (2010). Annual report to the nation on the status of cancer, 1975-2006, featuring colorectal cancer trends and impact of interventions (risk factors, screening, and treatment) to reduce future rates. Cancer.

[3] Tebbutt NC (2003). Intestinal complications after chemotherapy for patients with unresected primary colorectal cancer and synchronous metastases. Gut.

[4] Ricci-Vitiani L (2007). Identification and expansion of human colon-cancer-initiating cells. Nature.

[5] Dallas NA (2009). Chemoresistant colorectal cancer cells, the cancer stem cell phenotype, and increased sensitivity to insulin-like growth factor-I receptor inhibition. Cancer Res..

[6] Horst D, Kriegl L, Engel J, Kirchner T, Jung A (2008). CD133 expression is an independent prognostic marker for low survival in colorectal cancer. Br. J. Cancer.

[7] Horst D, Kriegl L, Engel J, Kirchner T, Jung A (2009). Prognostic significance of the cancer stem cell markers CD133, CD44, and CD166 in colorectal cancer. Cancer Invest..

[8] Merlos-Suarez A (2011). The intestinal stem cell signature identifies colorectal cancer stem cells and predicts disease relapse. Cell Stem Cell.

[9] Huang YF (2017). Curcumin enhances the effects of irinotecan on colorectal cancer cells through the generation of reactive oxygen species and activation of the endoplasmic reticulum stress pathway. Oncotarget.

[10] Zhu D. J., et al. Curcumin partly ameliorates irinotecan-induced diarrhea and synergistically promotes apoptosis in colorectal cancer through mediating oxidative stress. *Oncotarget*10.8632/oncotarget.10604 (2016).

[11] Zhu DJ (2014). Proteomic analysis identifies proteins associated with curcumin-enhancing efficacy of irinotecan-induced apoptosis of colorectal cancer LOVO cell. Int. J. Clin. Exp. Pathol..

[12] Kurtz A, Wang HL, Darwiche N, Harris V, Wellstein A (1997). Expression of a binding protein for FGF is associated with epithelial development and skin carcinogenesis. Oncogene.

[13] Schulze D, Plohmann P, Hobel S, Aigner A (2011). Anti-tumor effects of fibroblast growth factor-binding protein (FGF-BP) knockdown in colon carcinoma. Mol. Cancer.

[14] Tassi E (2006). Expression of a fibroblast growth factor–binding protein during the development of adenocarcinoma of the pancreas and colon. Cancer Res..

[15] Wilson PM, Ladner RD, Lenz HJ (2007). Predictive and prognostic markers in colorectal cancer. Gastrointest. Cancer Res..

[16] Apps MG, Choi EH, Wheate NJ (2015). The state-of-play and future of platinum drugs. Endocr. Relat. Cancer.

[17] Siegmund KD, Marjoram P, Woo YJ, Tavare S, Shibata D (2009). Inferring clonal expansion and cancer stem cell dynamics from DNA methylation patterns in colorectal cancers. Proc. Natl Acad. Sci. USA.

[18] Song L, Li Y (2016). The role of stem cell DNA methylation in colorectal carcinogenesis. Stem Cell Rev..

[19] Meredith GD (2015). Glycogen synthase kinase-3 (Gsk-3) plays a fundamental role in maintaining DNA methylation at imprinted loci in mouse embryonic stem cells. Mol. Biol. Cell.

[20] Popkie AP (2010). Phosphatidylinositol 3-kinase (PI3K) signaling via glycogen synthase kinase-3 (Gsk-3) regulates DNA methylation of imprinted loci. J. Biol. Chem..

[21] Donnenberg VS, Donnenberg AD (2005). Multiple drug resistance in cancer revisited: the cancer stem cell hypothesis. J. Clin. Pharmacol..

[22] Jeter CR (2011). NANOG promotes cancer stem cell characteristics and prostate cancer resistance to androgen deprivation. Oncogene.

[23] Kitahara T (2017). Identification and characterization of CD107a as a marker of low reactive oxygen species in chemoresistant cells in colorectal cancer. Ann. Surg. Oncol..

[24] Li L (2012). Activation of p53 by SIRT1 inhibition enhances elimination of CML leukemia stem cells in combination with imatinib. Cancer Cell.

[25] Prabhu VV (2016). Small-molecule prodigiosin restores p53 tumor suppressor activity in chemoresistant colorectal cancer stem cells via c-Jun-mediated DeltaNp73 inhibition and p73 activation. Cancer Res..

[26] Feng Y (2012). EGF signalling pathway regulates colon cancer stem cell proliferation and apoptosis. Cell Prolif..

[27] Wang YK (2010). Activation of Akt and MAPK pathways enhances the tumorigenicity of CD133+primary colon cancer cells. Carcinogenesis.

[28] Tassi E (2001). Enhancement of fibroblast growth factor (FGF) activity by an FGF-binding protein. J. Biol. Chem..

[29] Xu S (2014). Proteomic analysis of the human cyclin-dependent kinase family reveals a novel CDK5 complex involved in cell growth and migration. Mol. Cell Proteomics.

[30] Bhoumik A (2008). Suppressor role of activating transcription factor 2 (ATF2) in skin cancer. Proc. Natl Acad. Sci. USA.

[31] Arcaroli JJ (2012). Common PIK3CA mutants and a novel 3’ UTR mutation are associated with increased sensitivity to saracatinib. Clin. Cancer Res..

[32] Moon BS (2014). Role of oncogenic K-Ras in cancer stem cell activation by aberrant Wnt/beta-catenin signaling. J. Natl Cancer Inst..

[33] Luchtenborg M (2005). Mutations in APC, CTNNB1 and K-ras genes and expression of hMLH1 in sporadic colorectal carcinomas from the Netherlands Cohort Study. BMC Cancer.

[34] Schweigert A (2016). Activation of the Wnt/beta-catenin pathway is common in Wilms tumor, but rarely through beta-catenin mutation and APC promoter methylation. Pediatr. Surg. Int..

[35] Shenoy AK (2012). Transition from colitis to cancer: high Wnt activity sustains the tumor-initiating potential of colon cancer stem cell precursors. Cancer Res..

[36] Koh TJ (2000). Gastrin is a target of the beta-catenin/TCF-4 growth-signaling pathway in a model of intestinal polyposis. J. Clin. Invest..

[37] Prieur A (2017). Targeting the Wnt pathway and cancer stem cells with anti-progastrin humanized antibodies: a major breakthrough for K-RAS mutated colorectal cancer treatment. Clin. Cancer Res..

[38] Song L, Li Y, He B, Gong Y (2015). Development of small molecules targeting the Wnt signaling pathway in cancer stem cells for the treatment of colorectal cancer. Clin. Colorectal Cancer.

[39] Dar AA, Belkhiri A, El-Rifai W (2009). The aurora kinase A regulates GSK-3beta in gastric cancer cells. Oncogene.

[40] Kim do Y (2016). A novel miR-34a target, protein kinase D1, stimulates cancer stemness and drug resistance through GSK3/beta-catenin signaling in breast cancer. Oncotarget.

[41] Leis H, Segrelles C, Ruiz S, Santos M, Paramio JM (2002). Expression, localization, and activity of glycogen synthase kinase 3beta during mouse skin tumorigenesis. Mol. Carcinog..

[42] Shakoori A (2007). Inhibition of GSK-3 beta activity attenuates proliferation of human colon cancer cells in rodents. Cancer Sci..

[43] Zhou A (2016). Nuclear GSK3beta promotes tumorigenesis by phosphorylating KDM1A and inducing its deubiquitylation by USP22. Nat. Cell Biol..

[44] Cai G, Wang J, Xin X, Ke Z, Luo J (2007). Phosphorylation of glycogen synthase kinase-3 beta at serine 9 confers cisplatin resistance in ovarian cancer cells. Int. J. Oncol..

[45] Sokolosky M (2014). Inhibition of GSK-3beta activity can result in drug and hormonal resistance and alter sensitivity to targeted therapy in MCF-7 breast cancer cells. Cell Cycle.

[46] Li Z, Tan F, Thiele CJ (2007). Inactivation of glycogen synthase kinase-3beta contributes to brain-derived neutrophic factor/TrkB-induced resistance to chemotherapy in neuroblastoma cells. Mol. Cancer Ther..

[47] Tan J (2005). Pharmacologic modulation of glycogen synthase kinase-3beta promotes p53-dependent apoptosis through a direct Bax-mediated mitochondrial pathway in colorectal cancer cells. Cancer Res..

[48] Abecassis I (2008). Re-expression of DNA methylation-silenced CD44 gene in a resistant NB4 cell line: rescue of CD44-dependent cell death by cAMP. Leukemia.

[49] Yi JM (2008). Abnormal DNA methylation of CD133 in colorectal and glioblastoma tumors. Cancer Res..

[50] Livak KJ, Schmittgen TD (2001). Analysis of relative gene expression data using real-time quantitative PCR and the 2(-Delta Delta C(T)) method. Methods.

