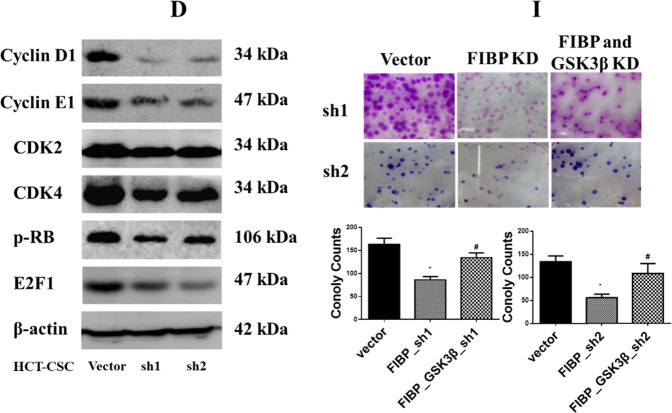# Correction: FIBP knockdown attenuates growth and enhances chemotherapy in colorectal cancer via regulating GSK3β-related pathways

**DOI:** 10.1038/s41389-022-00407-5

**Published:** 2022-06-24

**Authors:** Yan-Feng Huang, Wen-Bo Niu, Rong Hu, Ling-Jun Wang, Zeng-Yan Huang, Shi-Hao Ni, Ming-Qing Wang, Yi Yang, Yu-Sheng Huang, Wen-Jun Feng, Wei Xiao, Da-Jian Zhu, Shao-Xiang Xian, Lu Lu

**Affiliations:** 1 The First Affiliated Hospital, Guangzhou University of Chinese Medicine, 510407 Guangzhou, Guangdong China; 2 Shunde Hospital (The first People’s Hospital of Shunde Foshan), Southern Medical University, 528300 Foshan, China; 3 Cancer Research Institute, Southern Medical University, 510515 Guangzhou, China; 4 School of Traditional Chinese Medicine, Southern Medical University, 510515 Guangzhou, China; 5 Lingnan Medical Research Center, Guangzhou University of Chinese Medicine, 510407 Guangzhou, Guangdong China; 6 Department of Gastrointestinal Surgery, Guangdong Medical University Affiliated Women and Children Hospital, 528300 Foshan, China

Correction to: *Oncogenesis* 10.1038/s41389-018-0088-9, published online 02 October 2018

We regret the errors in Figure 3D and Figure 6I during typesetting. The figures below are corrected. In addition, the picture of flow cytometry in Figure S1A (After gating) and Figure 4D (Vector) is identical. This is because they do come from the same sample. This sample is set as a workflow sample in Figure S1A and set as control in Figure 4D. The workflow sample in Figure S1A were routinely treat with non-fluorescent vector. The changes do not affect the conclusion of the article. We apologies for any inconvenience caused.

The corrected panels in Figure 3D and Figure 6I.