# Supplementary material for: FIBP knockdown attenuates growth and enhances chemotherapy in colorectal cancer via regulating GSK3β-related pathways
Source: Oncogenesis. 2018 Oct 2;7(9):77. doi: 10.1038/s41389-018-0088-9 (PMC6167373; doi:10.1038/s41389-018-0088-9)
Supplement: Supplementary file 1 — SUPPLEMENTAL Figures 1-9 [file 41389_2018_88_MOESM1_ESM.docx]

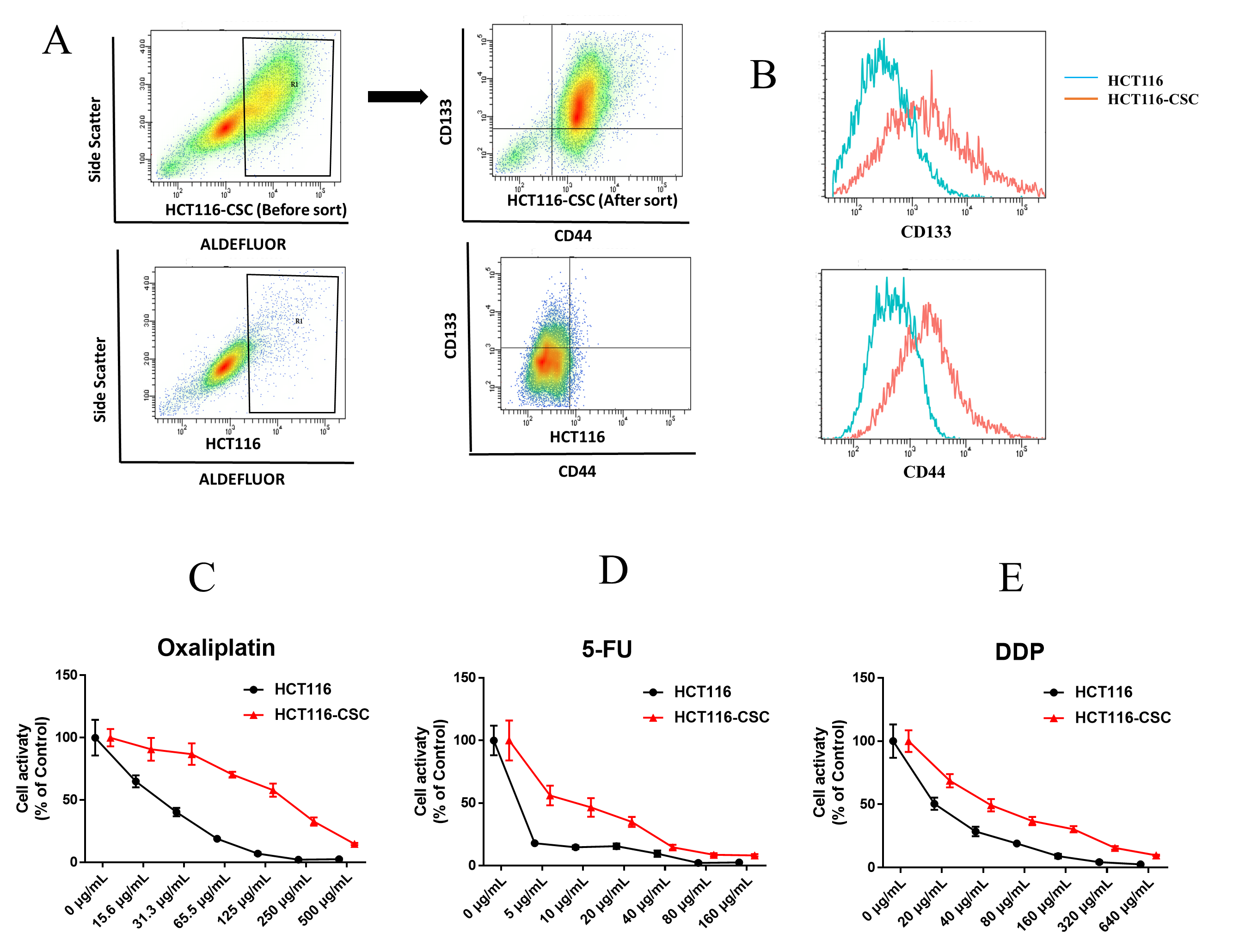


Figure S1. Identification of the chemoresistant HCT116-CSC cell line. (A) The workﬂow and FACS gating strategy for separation of cancer stem cells, HCT116-CSC is a cell line with oxaliplatin-resistance. Representative flow cytometry profile showing the surface expression of the stem cell markers CD44 and CD133 in parental HCT116 and HCT116-CSC cell lines. (B) Representative histogram showing the significantly up-regulated expression of CD133 and CD44 in HCT116-CSCs. (C-E) HCT116-CSCs demonstrated chemoresistance to oxaliplatin (C), 5-FU (D) and cisplatin (E) according to a cell viability test. The data shown are representative results from 3 independent experiments.


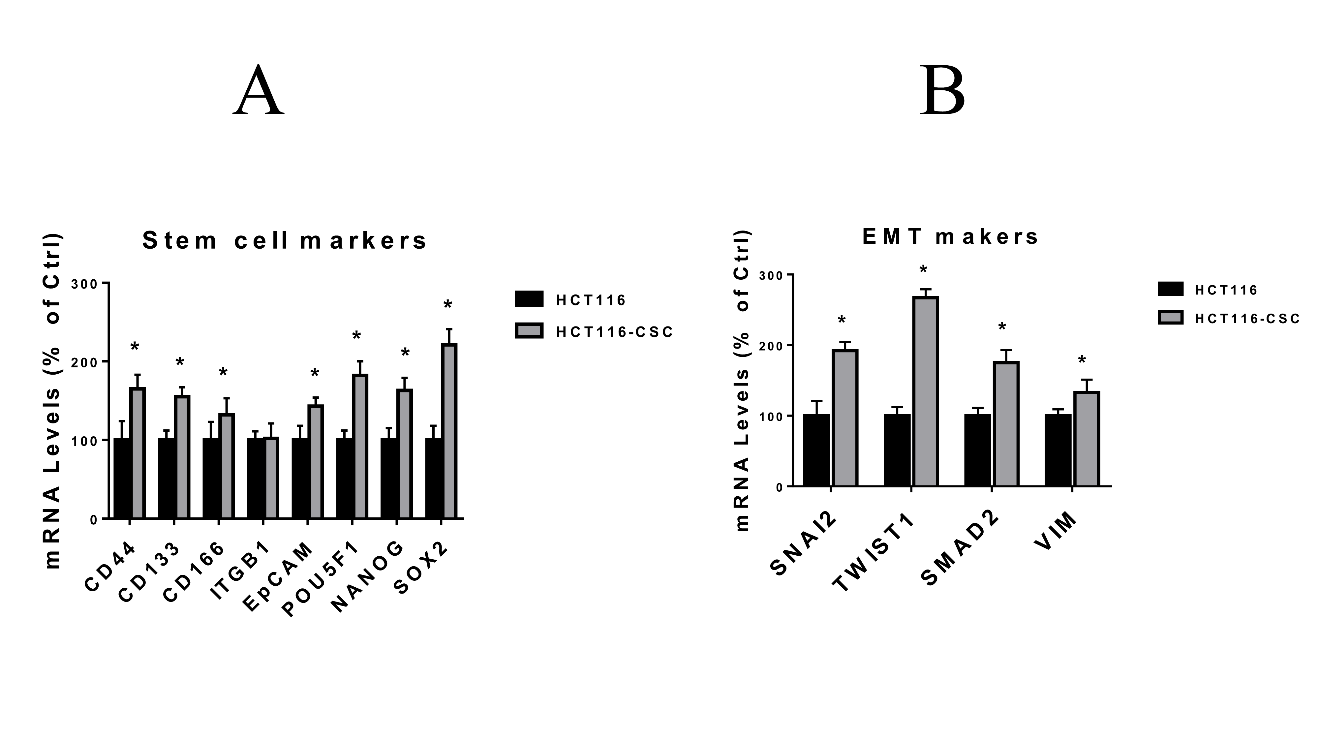


Figure S2. The mRNA levels of stem cell markers and EMT markers in parental HCT116 and HCT116-CSC cell lines. Seven stem cell markers, CD44, CD133, CD166, EpCAM, POU5F1, NANOG and SOX2 (A), and four EMT markers, SNAI2, TWIST1 SMAD2 and VIM (B), were quantitated by qPCR. n=8 for technical repeats in the qPCR assay. * p<0.05 compared with the parental HCT116 group.


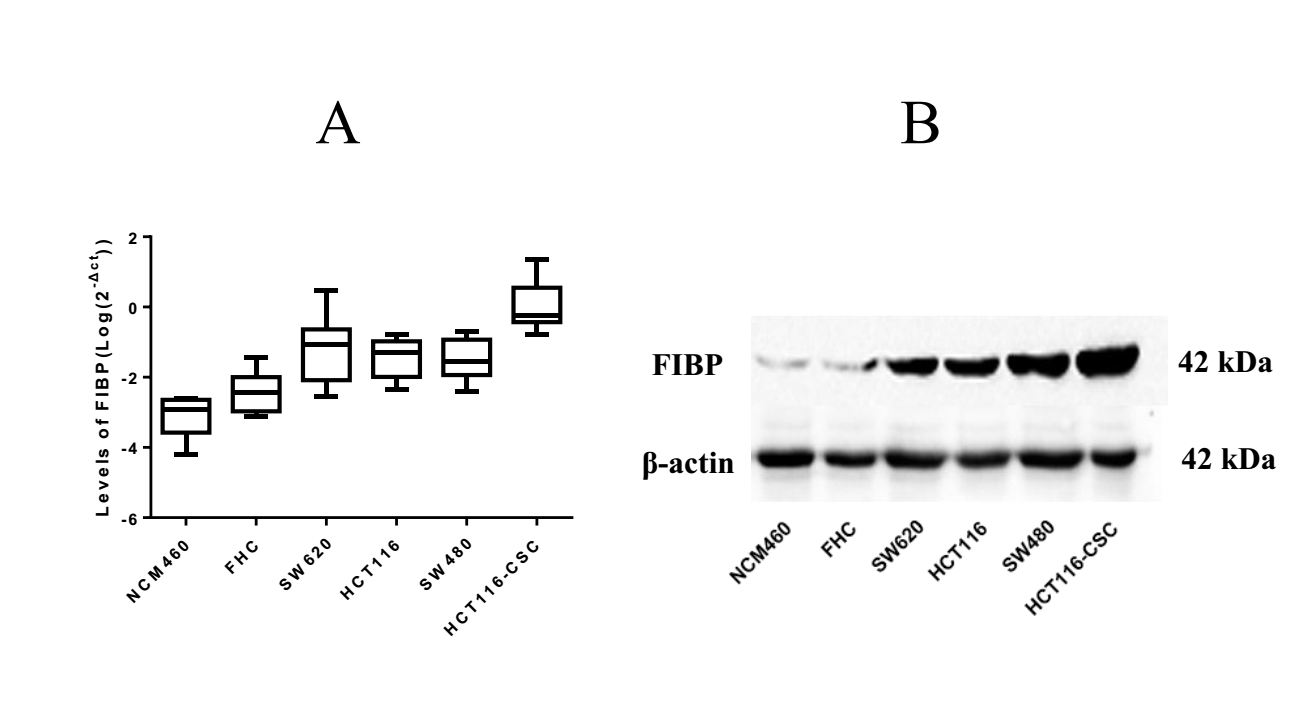


Figure S3. The expression levels of FIBP in normal human colonic epithelial cell lines and CRC cell lines. (A-B), mRNA levels of FIBP measured by qPCR (A) and protein levels of FIBP measured by western blot (B) in normal human colonic epithelial cell lines (NCM460 and FHC) and CRC cell lines (SW620, HCT116, SW480 and HCT116-CSC). n=8 for technical repeats in the qPCR assay, and the western blotting results shown represent one of three independent experiments.


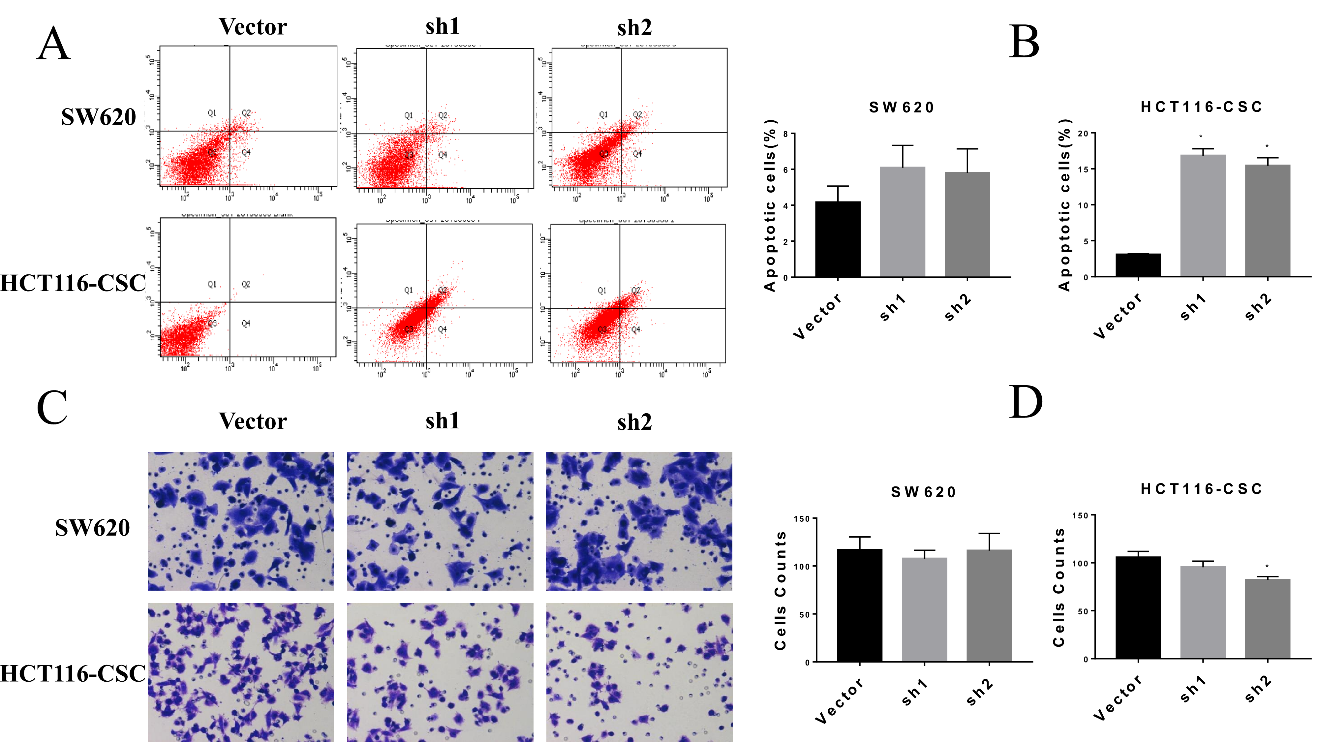


Figure S4. The effects of FIBP knockdown on apoptosis and migration of SW620 and HCT116-CSC cells. (A) Representative flow cytometry profile showing cell apoptosis in SW620 and HCT116-CSC cells with/without FIBP knockdown. (B) Summary results of the quantitation of cell apoptosis data. n=8 for each group. (C) Representative images of the migration assay of SW620 and HCT116-CSC cells with/without FIBP knockdown. (D) Summary results of the quantitation of cell migration data. * p<0.05 compared with the Vector control group.


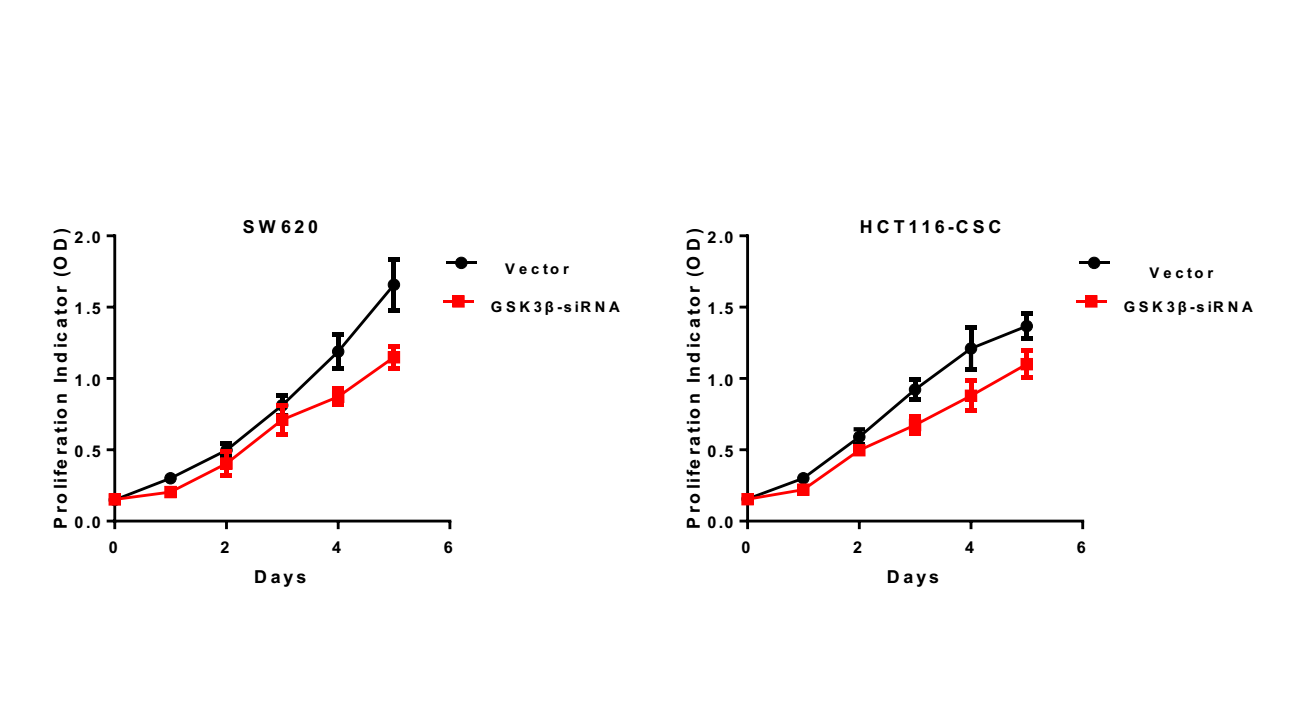


Figure S5. GSK3β knockdown inhibited the proliferation of both the SW620 (left) and HCT116-CSC (right) cell lines.


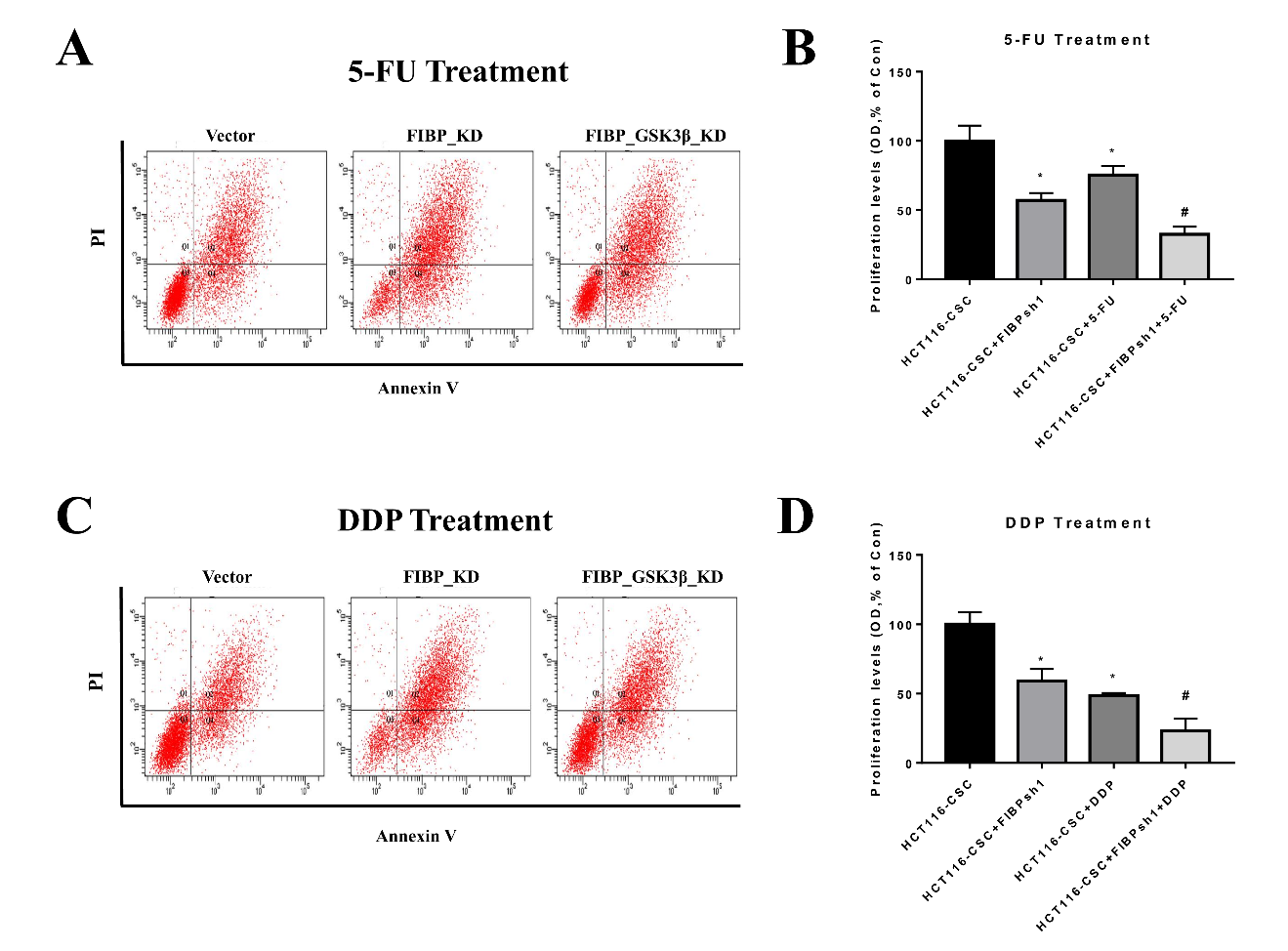


Figure S6. FIBP knockdown sensitized chemoresistant HCT116-CSCs. (A) Representative flow cytometry profile showing apoptosis of control HCT116-CSCs, HCT116-CSCs with FIBP knockdown alone and with combined knockdown of FIBP and GSK3β in the presence of 20 μg/ml 5-FU during culture. (B) The proliferation of HCT116-CSCs with/without FIBP knockdown in the absence or presence of 20 μg/ml 5-FU during culture. (C) Representative flow cytometry profile showing apoptosis of control HCT116-CSCs, HCT116-CSCs with FIBP knockdown alone and with combined knockdown of FIBP and GSK3β in the presence of 100 μg/ml DDP during culture. (D) The proliferation of HCT116-CSCs with/without FIBP knockdown in the absence or presence of 100 μg/ml DDP during culture. * p<0.05 compared with the Vector control group. # p<0.05 compared with the FIBP-shRNA group.


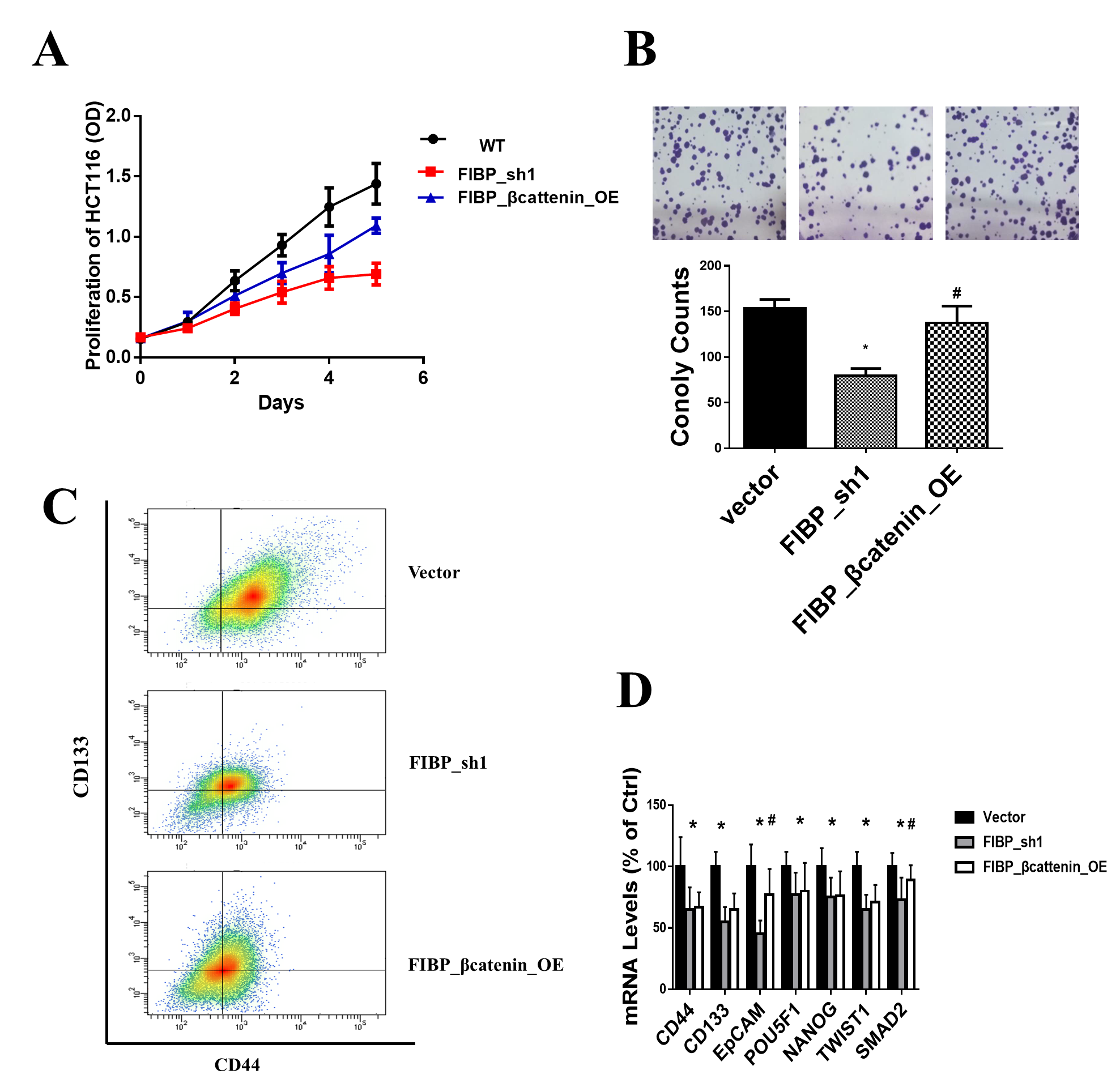


Figure S7. Over-expression of β-catenin restored HCT116-CSC cell proliferation after FIBP knockdown but did not reverse the reduced expression of stemness markers. (A) Additional overexpression of β-catenin restored the proliferation of HCT116-CSCs with FIBP knockdown. (B) Colony formation assay of HCT116-CSCs infected with control Vector lentivirus, FIBP-targeting shRNA lentivirus, and combined infection with FIBP-targeting shRNA lentivirus and transfection with β-catenin-expression plasmid. (C) The surface expression of CD44/CD133 decreased after FIBP knockdown and was not reversed by restoring β-catenin expression. (D) The mRNA levels of stem cell markers in HCT116-CSCs infected with control Vector lentivirus, FIBP-targeting shRNA lentivirus, and combined infection with FIBP-targeting shRNA lentivirus and transfection with β-catenin-expression plasmid. * p<0.05 compared with the Vector group. # p<0.05 compared with the FIBP-shRNA group.


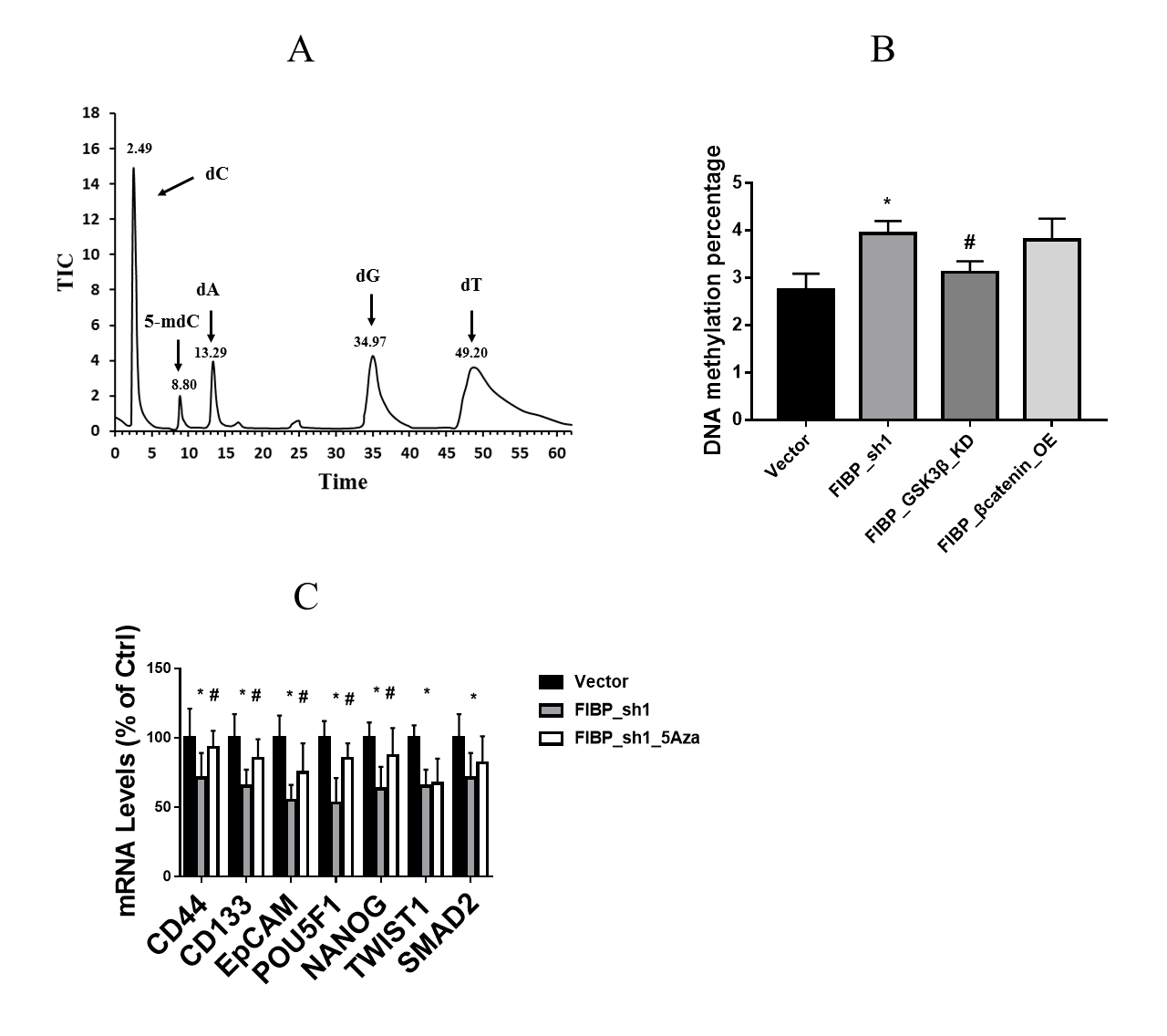


Figure S8. HPLC analysis of 5-mdC levels in HCT116-CSCs with different FIBP, GSK3β and β-catenin expression statuses. (A) Representative HPLC image showing the identification of chromatographic peaks for different deoxynucleoside samples. (B) The levels of 5mdC increased after FIBP knockdown, and the increase was reversed by additional GSK3β knockdown but not by overexpression of β-catenin. (C) The mRNA levels of stem cell markers in HCT116-CSCs infected with control Vector lentivirus, FIBP-targeting shRNA lentivirus, and combined infection with FIBP-targeting shRNA lentivirus and treatment with 5-aza-2′-deoxycytidine (5-Aza). * p<0.05 compared with the control Vector group. # p<0.05 compared with the FIBP-shRNA group.


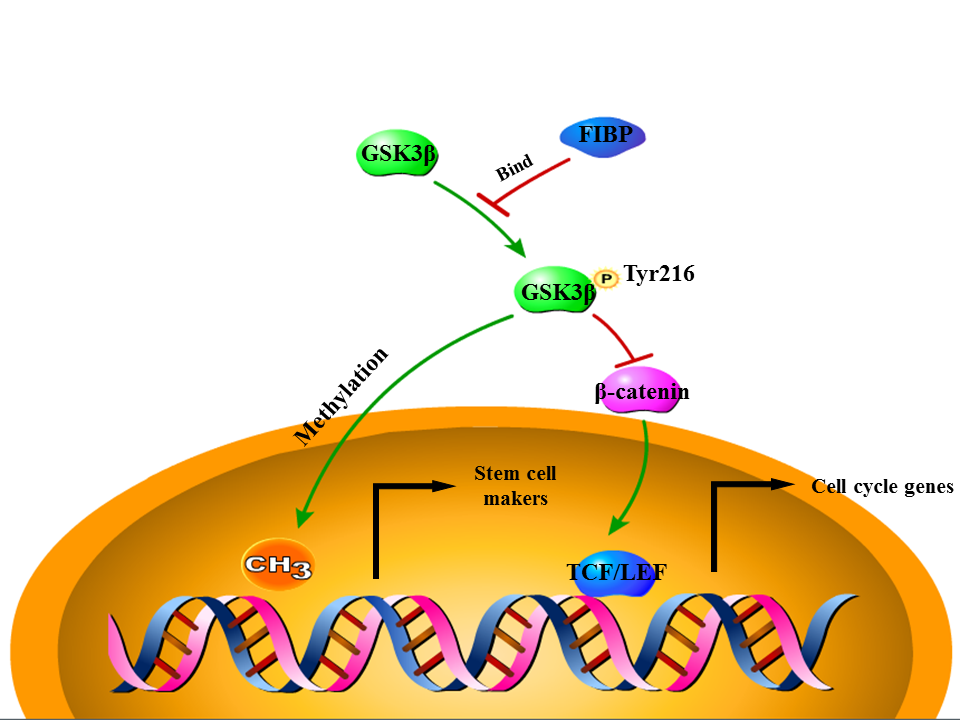


Figure S9. Proposed working model of FIBP-regulated proliferation and stem cell makers via modulation of GSK3β-controlled β-catenin/cyclin D1 signaling and methylation of stemness genes in CRC cells.
