# Supplementary material for: FIBP knockdown attenuates growth and enhances chemotherapy in colorectal cancer via regulating GSK3β-related pathways
Source: Oncogenesis. 2018 Oct 2;7(9):77. doi: 10.1038/s41389-018-0088-9 (PMC6167373; doi:10.1038/s41389-018-0088-9)
Supplement: Supplementary file 2 — SUPPLEMENTAL Tables 1-3 [file 41389_2018_88_MOESM2_ESM.docx]

**Table S1. Sequences of primers and short RNA oligos**

| **Gene** | **Description** | **Sequence** |
| --- | --- | --- |
| **FIBP** | Vector | 5’-TG TCTCGCTTGGGCGAGAGTA TTCAAGAGA TACTCTCGCCCAAGCGAGA CTTTTTTC-3’ |
|  | shRNA1 | 5’-TG TGCTGAATATTGTCCACCA TTCAAGAGA TGGTGGACAATATTCAGCA CTTTTTTC-3’ |
|  | shRNA2 | 5’-TG TTGGTGCTGATGTCATCCA TTCAAGAGA TGGATGACATCAGCACCAA CTTTTTTC-3’ |
|  | Primers for qRT-PCR | F: 5-CTTCCAGATTCCGCCCTCC-3 |
|  |  | R: 5- AGCACCTCCCGAACAAAGG-3 |
| **CCND1** | Primers for amplification (NM_053056.2) | F: 5-CG GAATTC ATGGAACACCAGCTCCTGTG-3 |
|  |  | R: 5- GC TCTAGA TCAGATGTCCACGTCCCGCAC-3 |
| **CTNNB1** | Primers for amplification (NM_001098209) | F: 5- G GATCC  ATGGCTACTCAAGCTGATTTGATGG-3 |
|  |  | R: 5- GC TCTAGA  CAGGTCAGTATCAAACCAGGCCAGC-3 |
| **GSK3β** | Vector | 5’-CUUUCCCGGGAAAGUUAAUUU-3’ |
|  | siRNA | 5’-GAAGAAAGAUGAGGUCUAUUU-3’ |
| **CD44** | Primers for qRT-PCR | F: 5-CTGCCGCTTTGCAGGTGTA-3 |
|  |  | R: 5- CATTGTGGGCAAGGTGCTATT-3 |
| **CD133** | Primers for qRT-PCR | F: 5- GGCCCAGTACAACACTACCAA-3 |
|  |  | R: 5- ATTCCGCCTCCTAGCACTGAA-3 |
| **CD166** | Primers for qRT-PCR | F: 5- ACTTGACGTACCTCAGAATCTCA-3 |
|  |  | R: 5- CATCGTCGTACTGCACACTTT-3 |
| **ITGB1** | Primers for qRT-PCR | F: 5- CCTACTTCTGCACGATGTGATG-3 |
|  |  | R: 5- CCTTTGCTACGGTTGGTTACATT-3 |
| **EpCAM** | Primers for qRT-PCR | F: 5- AATCGTCAATGCCAGTGTACTT-3 |
|  |  | R: 5- TCTCATCGCAGTCAGGATCATAA-3 |
| **POU5F1** | Primers for qRT-PCR | F: 5- CTGGGTTGATCCTCGGACCT-3 |
|  |  | R: 5- CCATCGGAGTTGCTCTCCA-3 |
| **NANOG** | Primers for qRT-PCR | F: 5- TTTGTGGGCCTGAAGAAAACT-3 |
|  |  | R: 5- AGGGCTGTCCTGAATAAGCAG-3 |
| **SOX2** | Primers for qRT-PCR | F: 5- GCCGAGTGGAAACTTTTGTCG-3 |
|  |  | R: 5- GGCAGCGTGTACTTATCCTTCT-3 |
| **SNAI2** | Primers for qRT-PCR | F: 5- CGAACTGGACACACATACAGTG-3 |
|  |  | R: 5- CTGAGGATCTCTGGTTGTGGT-3 |
| **TWIST1** | Primers for qRT-PCR | F: 5- GTCCGCAGTCTTACGAGGAG-3 |
|  |  | R: 5- GCTTGAGGGTCTGAATCTTGCT-3 |
| **SMAD2** | Primers for qRT-PCR | F: 5- CCGACACACCGAGATCCTAAC-3 |
|  |  | R: 5- GAGGTGGCGTTTCTGGAATATAA-3 |
| **VIM** | Primers for qRT-PCR | F: 5- GACGCCATCAACACCGAGTT-3 |
|  |  | R: 5- CTTTGTCGTTGGTTAGCTGGT-3 |
| **GAPDH** | Primers for qRT-PCR | F: 5- GGAGCGAGATCCCTCCAAAAT -3 |
|  |  | R: 5- GGCTGTTGTCATACTTCTCATGG -3 |

**Table S2: Crude and adjusted hazard risk for FIBP expression and its association with CRC patient survival**

| **Parameter** | **Univariate analysis** | | | **Multivariate analysis** | | |
| --- | --- | --- | --- | --- | --- | --- |
|  | **HR** | **95% CI** | **p value** | **HR** | **95% CI** | **p value** |
| **Age (>65 yr *vs.* ≤65 yr)** | 1.233 | 0.639~2.378 | 0.532 | 1.473 | 0.743~2.921 | 0.267 |
| **Gender (male *vs.* female)** | 0.666 | 0.347~1.278 | 0.222 | 0.540 | 0.275~1.058 | 0.073 |
| **TNM stage (III+IV *vs.* I+II)** | 2.611 | 1.191~5.723 | 0.017* | 1.785 | 0.789~4.040 | 0.164 |
| **Tumor size (>5 cm *vs.* ≤5 cm)** | 1.560 | 0.818~2.974 | 0.177 | 1.309 | 0.672~2.550 | 0.429 |
| **AJCC stage (III+IV *vs.* I+II)** | 2.613 | 1.365~5.001 | 0.004* | 3.395 | 1.667~6.913 | 0.001* |
| **FIBP expression (high *vs.* low)** | 2.696 | 1.407~5.166 | 0.003* | 2.988 | 1.512~5.903 | 0.002* |

**Note: 8 samples are missing due to drop off; *, p<0.05. These parameters were adjusted by a Cox regression in multivariate analysis.**

**HR: hazard ratio; CI: confidence interval; TNM:** **Tumor Node Metastasis; AJCC:** **American Joint Committee on Cancer**

**Table S3: Methylation status of genes involved in regulating CRC cell stemness and EMT.**

| **Array ID** | **Seq names** | **Start** | **End** | **Symbol** | **Beta-value** | | |
| --- | --- | --- | --- | --- | --- | --- | --- |
|  |  |  |  |  | **Vector** | **FIBP_KD** | **FIBP/GSK3b_KD** |
| **cg01663768** | chr11 | 35160844 | 35160966 | CD44 | 0.257 | 0.948 | 0.222 |
| **cg03101849** | chr4 | 16084683 | 16084805 | CD133 (PROM1) | 0.152 | 0.82 | 0.164 |
| **cg03376719** | chr3 | 105086941 | 105087063 | CD166 (ALCAM) | 0.166 | 0.421 | 0.289 |
| **cg04276417** | chr10 | 33227910 | 33228032 | ITGB1 | 0.874 | 0.684 | 0.869 |
| **cg06233293** | chr2 | 47596186 | 47596308 | EPCAM | 0.266 | 0.921 | 0.489 |
| **cg06576021** | chr8 | 49830938 | 49831060 | SNAI2 | 0.896 | 0.913 | 0.963 |
| **cg09674215** | chr7 | 19158135 | 19158257 | TWIST1 | 0.85 | 0.941 | 0.915 |
| **cg16131053** | chr18 | 45459277 | 45459399 | SMAD2 | 0.812 | 0.915 | 0.844 |
| **cg19111999** | chr10 | 17270088 | 17270210 | VIM | 0.858 | 0.892 | 0.862 |
| **cg19204662** | chr6 | 31139278 | 31139400 | POU5F1 | 0.885 | 0.914 | 0.804 |
| **cg00774437** | chr12 | 7939675 | 7939797 | NANOG | 0.81 | 0.879 | 0.748 |
| **cg19462523** | chr3 | 181429909 | 181430031 | SOX2 | 0.474 | 0.871 | 0.927 |
